# Anisotropic wear behavior of meniscus: Influence of cross-shear and loading magnitude

**DOI:** 10.1016/j.jmbbm.2025.107212

**Published:** 2025-09-23

**Authors:** Kate J. Benfield, Katherine J. Fors, Trevor C. Black, Giada A. Brandes, Karlee M. Macaw, Vanessa Bowman, Cynthia Keller-Peck, Trevor J. Lujan

**Affiliations:** a Biomedical Engineering Doctoral Program, Boise State University, Boise, ID, USA; b Department of Mechanical & Biomedical Engineering, Boise State University, Boise, ID, USA; c Biomolecular Research Center, Boise State University, Boise, ID, USA

**Keywords:** Meniscus degeneration, Musculoskeletal biomechanics, Tribology, Fibrillation, Fatigue testing, 3D optical scanning

## Abstract

The repetitive wear-and-tear of knee menisci contributes to chronic knee pain and disability, yet the mechanical factors driving this degenerative process are poorly understood. Here we characterize the effect of motion type and loading magnitude on the anisotropic wear behavior of bovine meniscus. Custom pin-on-plate systems applied 60,000 cycles of unidirectional motion or multidirectional (cross-shear) motion by translating a sectioned “plate” of meniscus under a fixed cartilage “pin” that was loaded to generate physiological stress conditions (0.5, 1.0, 1.5 MPa). Pin motion was applied either longitudinal or transverse to the circumferential fibers of the meniscal tissue. We measured the effect of wear testing on meniscal volume loss, compressive mechanical properties, fiber fraying, and superficial layer thickness. A three-fold increase in loading magnitude resulted in a 36% increase in volume loss and a significant increase in fiber fraying. Multidirectional motion resulted in 31% greater volume loss than unidirectional motion, however, this change was not significant. Transverse specimens exhibited 1.8x greater volume loss than longitudinal specimens. Multiple regression revealed that meniscal tissue was more resistant to wear when it had higher initial tissue stiffness and greater initial stress relaxation. For the first time, this study has demonstrated that the meniscus exhibits anisotropic wear behavior that is governed by the compressive loading magnitude. This study provides foundational data and mechanistic insights on the wear behavior of the knee meniscus.

## 1. Introduction

The repetitive wear-and-tear of knee menisci plays a significant role in chronic knee pain and disability, yet little is understood about the mechanical factors that drive this insidious degenerative process. Knee menisci are fibrocartilaginous structures that facilitate smooth articulations and provide joint stability while distributing and attenuating 50–70% of contact forces across the knee joint (Fox et al., 2012; Ahmed and Burke, 1983). The load bearing capacity of the meniscus is dictated by its highly organized collagen fiber network, where type I collagen fibers in the internal central layer exhibit anisotropy by orienting along the circumferential direction and in the superficial layer exhibit isotropy by orienting randomly (Fox et al., 2012; Gelbart and Firer, 2009). With age and overuse, the menisci become prone to degeneration – a retrogressive breakdown of the collagen fibers that results in extracellular matrix (ECM) weakening, fiber fraying and delamination, and a loss of proteoglycans and chondrocytes (Warnecke et al., 2020; Pauli et al., 2011; Tsujii et al., 2017). Meniscal degeneration is highly prevalent, affecting nearly 20% of individuals over the age of 50 years and more than 55% of those over 70 years of age (Englund et al., 2008). Degenerative changes to the meniscal ECM can cause knee joint instability, increased risk of multiplanar tears, pain, and a higher likelihood of developing knee osteoarthritis (OA) (Gelbart and Firer, 2009; Pauli et al., 2011; Englund et al., 2008; Howell et al., 2014).

While extensive research has been dedicated to elucidating the biological pathways underlying this degeneration (i.e. inflammation, production of ECM degrading enzymes, chondrocyte hypertrophy) (Bradley et al., 2023; Fang and Lake, 2017; Huang et al., 2021; Nguyen et al., 2017), comparatively less attention has been given to a direct mechanical pathway leading towards degeneration, independent of biological responses. Addressing this gap is essential for a comprehensive understanding of disease pathomechanics and for developing effective preventative strategies to reduce meniscal degeneration. Currently, efforts to understand such mechanical pathways have predominately focused on articular cartilage and its association with OA, where studies have shown that repeated articular cartilage exposure to abnormal loads results in softening at the molecular level, structural damage, tissue wear, and eventual dysfunction (Hosseini et al., 2013; Lizhang et al., 2011; Moore and Burris, 2015; Petersen et al., 2023; Vazquez et al., 2019). However, recent evidence has suggested that meniscal degeneration may actually precede cartilage degradation in the onset of OA (Seitz et al., 2021). This raises the possibility that meniscal degeneration plays a primary role in the onset of joint degeneration, thus emphasizing the need to investigate the underlying mechanical factors of meniscal degeneration, including mechanical wear.

Mechanical wear is a system response dependent upon contact conditions between interfacing surfaces (Bayer, 2004). It is most commonly quantified by calculating material mass or volume loss (Bayer, 2004) through methods such as gravimetric analysis, micro-CT imaging, and more recently, 3D optical scanning techniques (Benfield et al., 2022; Elsner et al., 2015; Hollar et al., 2018). Several in vitro studies have utilized various tribological testing configurations (i.e. pendulum systems, pin-on-disc, pin-on-plate devices) to conduct accelerated wear tests between lubricated, small-sectioned biomaterials with controllable contact conditions (i.e. contact stress, sliding speed, lubricant) (Hossain et al., 2020; Northwood and Fisher, 2007; Majd et al., 2017; Oungoulian et al., 2015; McCann et al., 2009). Such studies have typically tested articular cartilage or meniscus samples against a glass, polyethylene, steel, or cartilage substrate with lubricants ranging from phosphate-buffered saline (PBS) to fetal bovine serum (FBS) (Link et al., 2020). These prior biotribological studies have quantified the coefficient of friction (COF) in healthy native articular cartilage to range between 0.001 and 0.46 (Lizhang et al., 2011; Moore and Burris, 2015; Link et al., 2020; Kanca et al., 2018) and 0.09 to 0.30 for meniscus (Majd et al., 2017; Link et al., 2020; Galley et al., 2011; Warnecke et al., 2017), where increases in COF are implicitly assumed to correspond with increases in wear. Although the COF for meniscal tissue has been reported, the biotribological nature of the native meniscus remains relatively unexplored, with no study characterizing anisotropic wear behaviors of the meniscus.

In the human knee joint, the menisci experience complex contact conditions including sliding motion paths and contact stresses (axial and torsional) (Mündermann et al., 2008; Reinders et al., 2015). Importantly, these contact conditions will vary in vivo based on many intrinsic (e.g. joint anatomy) and extrinsic (e.g. motion type, loading magnitude) factors. For instance, different activities create distinct in-plane joint translations and joint rotations between the cartilage and meniscus. These multidirectional motion paths generate environments of continuously changing shear, or cross-shear. Numerous in vitro tribological studies have shown this cross-shear to accelerate wear in polyethylene joint replacement components (Abdelgaied et al., 2018; Bragdon et al., 1996; Kang et al., 2008; Turell et al., 2003), with wear rates up to two orders of magnitude greater than those under unidirectional motion (Bragdon et al., 1996). Similar cross-shearing mechanisms may also be a key mechanical factor in meniscal wear, as activities associated with multidirectional motion may compromise the meniscal fibrous network and, consequently, its structural integrity. For example, stair climbing creates multidirectional joint motions (Reinders et al., 2015; Schwiesau et al., 2013), and repeated stair climbing has been associated with nearly 2x greater risk of meniscal degeneration (Snoeker et al., 2013). Yet surprisingly, no study has directly examined cross-shearing as a mechanical factor for meniscal degeneration. Moreover, in addition to motion path, the magnitude of applied loading may be a driving mechanical factor influencing meniscal wear, as in vivo contact stresses are influenced by body weight, body-borne loads, and specific activity (Reinders et al., 2015; Willy et al., 2016). In comparison to level walking, knee joint forces are 3x greater when running (Miller et al., 2015) and nearly 2x higher during deep squatting (Schwiesau et al., 2013). However, the specific sensitivity of meniscal wear resistance to variations in applied loading magnitude remains poorly understood.

Understanding how such biomechanical factors contribute directly to meniscal wear and fraying can inform evidence-based strategies (e.g. exercise, diet, movement techniques) to prevent and delay knee degeneration, and ultimately OA. Therefore, the objective of this study was to characterize the effect of motion path and loading magnitude on meniscal wear behavior while accounting for fiber orientation of the internal central layer. Our hypothesis is that multidirectional motion between bovine cartilage-meniscus interfaces at high loading magnitudes will lead to significantly greater meniscal volume loss, tissue weakening, and fiber fraying.

## 2. Methods

### 2.1. Overview

A series of experiments were conducted using pin-on-plate test systems to characterize the in-vitro mechanical wear behavior of bovine meniscus under highly specific contact conditions: motion type (unidirectional, multidirectional), compressive loading magnitude (0.5, 1.0, 1.5 MPa), and fiber orientation (longitudinal, transverse). Meniscal volume loss was analyzed at the superficial surface using our novel 3D optical scanning techniques. Meniscus integrity was assessed using dynamic mechanical analysis (DMA) to evaluate changes in compressive properties before and after testing, while brightfield and polarized light microscopy were used to evaluate fiber fraying and superficial layer thickness, respectively.

### 2.2. Specimen preparation

The medial and lateral menisci were extracted from healthy, skeletally mature bovine stifle joints (Wakefield Meats, Kuna, ID) and sectioned into anterior and posterior regions. Each meniscal specimen underwent visual inspection to confirm the absence of macroscopic damage. Any specimen exhibiting visible defects was excluded. The natural curvature of each sectioned meniscus was flattened to ensure a uniform surface for conducting pin-on-plate wear tests (Bonomo et al., 2020). Additional details regarding the meniscal flattening can be found in the Supplemental Material, however, it is important to note that pilot DMA testing showed minimal differences in compressive properties between flattened and un-flattened tissue (*p* > 0.48), indicating that the flattening process did not grossly impact tissue mechanical behavior. Once flattened, the menisci were packed in cellu-clay and layered into ~2.5 mm thick strips following our established methods (Wale et al., 2021). Caution was taken to ensure specimens were not extracted near the meniscal roots, as tissue from these regions may not accurately represent the mechanical behavior of the meniscal body (Ellman et al., 2014). After layering, the superficial meniscal layers were cut into rectangles (25 mm × 15 mm × 2.5 mm) to serve as the “plate” during pin-on-plate wear experiments. This was done by aligning a custom punch along either the circumferential fiber direction (longitudinal group) or perpendicular to the circumferential fiber direction (transverse group). As the fiber network of the superficial layer is less organized than the inner layers (Fox et al., 2012), we distinguished between longitudinal and transverse fiber orientations by imaging the underside of the meniscal plate (i.e. top-most inner layers) using a benchtop microscope (Leica, DM750). Fiber orientation was verified using Fast-Fourier Transformation analyses in FiberFit software (Morrill et al., 2016) resulting in mean fiber orientations of 2.3 ± 1.6° and 91 ± 2.5° relative to the primary circum0066erential fiber orientation for longitudinal and transverse specimens, respectively.

For the pin, cartilage plugs were extracted from the femoral surface of the stifle joints using a drill press. Pins were extracted from regions of the medial and lateral femoral condyles such that the articular surface was perpendicular to the coring axis. This approach helped preserve a consistent, flat contacting area. Pins from the medial and lateral femoral condyles were paired with the medial and lateral menisci, respectively, to maintain proper anatomical matching. Each cartilage pin was 4 mm in diameter and composed of the cartilage layer (1.3 ± 0.1 mm) and approximately 10 mm of subchondral bone used for mounting. Meniscal plates were wrapped in gauze soaked in phosphate-buffer saline (PBS; 10 mM phosphate, 150 mM sodium chloride) and frozen (−4° C). On the day of testing, each specimen was thawed for 1h (room temperature) and allowed to soak in a PBS bath for an additional hour prior to imaging and mechanical testing (Benfield et al., 2022).

### 2.3. Pin-on-plate test systems for simulating mechanical wear

**Fig. 1.**
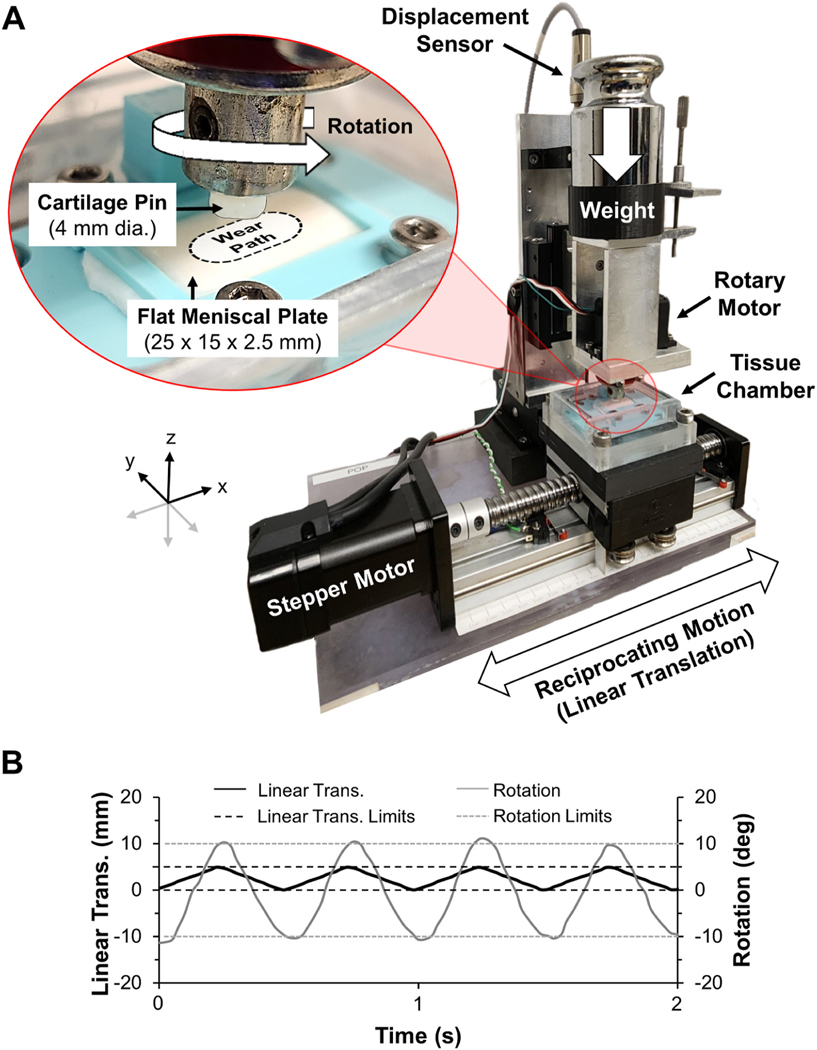
Custom pin-on-plate systems A) applied reciprocating linear motion and rotary motion to create cross-shear loading paths between a cartilage pin and meniscal plate B) at a synchronized frequency of 2.0 Hz.

Three custom pin-on-plate test systems were built for the purpose of characterizing meniscal wear under varying contact conditions (Fig. 1A). Reciprocating motion was achieved using a closed-loop stepper motor system (NEMA 17; CL42T driver) that utilized an encoder to continuously monitor and correct its position (5 mm stroke; translational accuracy = 20 ± 10 μm). The rotational component for multidirectional motion came from a servo rotary motor with position feedback (Pololu; FT1117M-FB) that enabled a closed-loop control of the servo (average pin-on-plate rotational accuracy = 1.3 ± 0.8° when applying 10°) (Fig. 1B). The stepper motor and servo motor were synchronized at a frequency of 2.0 Hz with a feedback sampling rate of 40 Hz for both motors (Fig. 1B). A high-resolution displacement sensor (Micro-Epsilon; DTA-3D-3-SA; accuracy ± 3 μm; sampling at 40 Hz) recorded the amount of meniscal displacement throughout testing. The stepper motor system and servo motor were controlled through a Teensy 4.1 microprocessor (PJRC; 600 MHz) while the displacement sensor was controlled through its own controller (Micro-Epsilon; MSC7401). Lab-VIEW software (National Instruments, v2017) facilitated communication between the Teensy, displacement sensor controller, and provided a graphical user interface. Additional information regarding the pin-on-plate test systems can be found in the Supplemental Material.

### 2.4. Meniscal wear testing

**Fig. 2.**
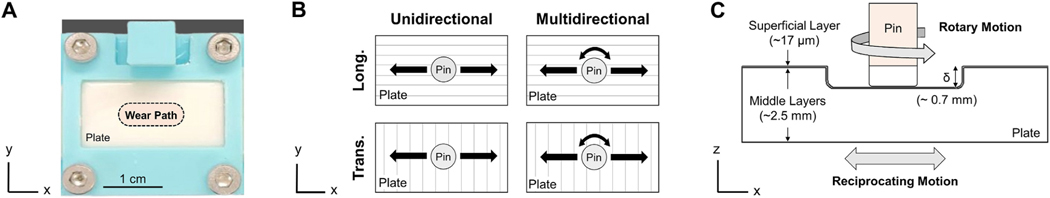
Wear testing was performed with the meniscal plate secured in A) a detachable test platform, where B) unidirectional and multidirectional motion was applied either longitudinal or transverse to the circumferential fiber orientation (gray lines). C) The reciprocating motion created a wear path with an average maximum displacement of 0.68 mm (δ), corresponding to a mean compressive strain of 23%.

Our pin-on-plate test systems (Fig. 1A) were used to examine three factors: motion type (unidirectional, multidirectional), compressive loading magnitude (0.5, 1.0, 1.5 MPa), and fiber orientation (longitudinal, transverse). Prior to testing, the meniscal plate was clamped into a detachable test platform (Fig. 2A), with friction tabs (i.e. sandpaper) adhered to the underside of the clamp to minimize tissue slippage (Wale et al., 2021). Each meniscal plate was positioned to align the circumferential fibers either longitudinally or transversely to the reciprocating linear motion (Fig. 2B). For unidirectional tests, the meniscal plate was translated relative to the fixed cartilage pin for 60, 000 cycles (5 mm stroke) (International Standard, 2014) while multidirectional tests incorporated a perpendicular component to the translational motion (cross-shear) by simultaneously rotating the cartilage pin (±10°) (Northwood and Fisher, 2007) (Fig. 1). Both motion types created a 4 mm × 11 mm wear path within the center of the meniscal plate (Fig. 2A), such that the contact area of the pin remained well within the boundaries of the clamped plate to avoid edge effects. Using weights, vertical loads of 6.3 N, 12.6 N, and 18.8 N were applied between the 4 mm diameter cartilage pin and superficial meniscal layer (~17 μm) interface (Fig. 2C). These forces corresponded to 0.5 MPa, 1.0 MPa, and 1.5 MPa, representing physiological stresses from walking to deep squatting (D’Lima et al., 2012; Gilbert et al., 2014). A pre-conditioning phase of 100 cycles was applied at the same load and frequency used for wear testing to mitigate initial tissue settling affects. During wear testing, the displacement sensor was used to calculate compressive strain as the change in meniscal thickness over its initial thickness (measured under a static 0.1 N load). Maximum compressive strain was calculated as the average strain from the last 1K cycles, and steady-state creep rate was calculated as the slope of the linear fit within the steady-state region (40K–60K cycles).

The three tested factors created 12 distinct wear groups resulting in a total of 72 wear tests conducted under a fully factorial design (*n* = 6 meniscal plates and *n* = 6 cartilage pins per group). Specimens within each group were acquired from different bovine stifle joints with an equal number of plate specimens from both the medial and lateral meniscus, as well as an equal number from the anterior and posterior meniscal regions. An additional 18 load soak controls were used to distinguish between viscoelastic creep and permanent deformation (compression only; longitudinal fiber orientation only; *n* = 6 per loading magnitude). Furthermore, an additional 6 unloaded controls, consisting of specimens with no applied motion and no loading (*n* = 6; longitudinal fiber orientation only), were also evaluated to serve as a baseline for assessing the effect of loading magnitude. This brought the total number of specimens tested to 96 (*n* = 72 wear; *n* = 18 load soak; *n* = 6 unloaded). All testing was done in a PBS bath with protease inhibitor lubricant (cOmplete^™^, Mini Protease Inhibitor Cocktail; Sigma-Aldrich) to prevent enzymatic degradation of the tissue during testing (Leung et al., 2000). While PBS lacks the complex biochemical components (e.g. proteins, lipids) of native synovial fluid (Ghosh et al., 2014), it is widely used in tribological studies as a baseline lubricant (Moore and Burris, 2015; Hossain et al., 2020; Oungoulian et al., 2015; Durney et al., 2020).

### 2.5. 3D optical scanning to quantify and visualize volume loss

To quantify and visualize volume loss, the meniscal plate was imaged via a 3D optical scanning system at three time points: before wear testing (pre-test), 5 min directly after wear testing (post-test), and following a 24 h unloaded recovery period in PBS plus protease solution (post-recovery) (Benfield et al., 2022, 2023). The 3D optical scanning system consisted of a rotary table, projector (HDI Advance R2 projector with 17.5 mm/F8 83954 lenses), and two high-resolution cameras (LMI Technologies, Delta, Canada; 2.8 megapixels) (Hollar et al., 2018). This system was operated with FlexScan3D software (LMI Technologies; v3.3.21.8), and calibrated using a calibration card consisting of a 2.5 × 2.0 cm grid of 2 × 2 mm black and white squares (Hollar et al., 2018). All scanning was performed in a dark room to minimize the presence of ambient light that could impact scanning quality (Benfield et al., 2023). The projector field of view (FOV) was approximately 25 cm^2^ (scanning accuracy = 2.7 μm). The exposure settings were set to high dynamic range (HDR), which automatically determined the optimal exposure (range of exposure times = 16.67–150 ms) (Benfield et al., 2022, 2023). The meniscal plate was scanned every 15° using the rotary table for a total of 8 scans resulting in a 120-degree 3D captured view of the meniscal surface. A low-lint Kimtech wipe (Kimtech Science, Kimberly-Clark) was used to lightly absorb any remaining PBS droplets that may have been present to reduce the potential of scanning artifacts and impaired scan quality (Benfield et al., 2022).

**Fig. 3.**
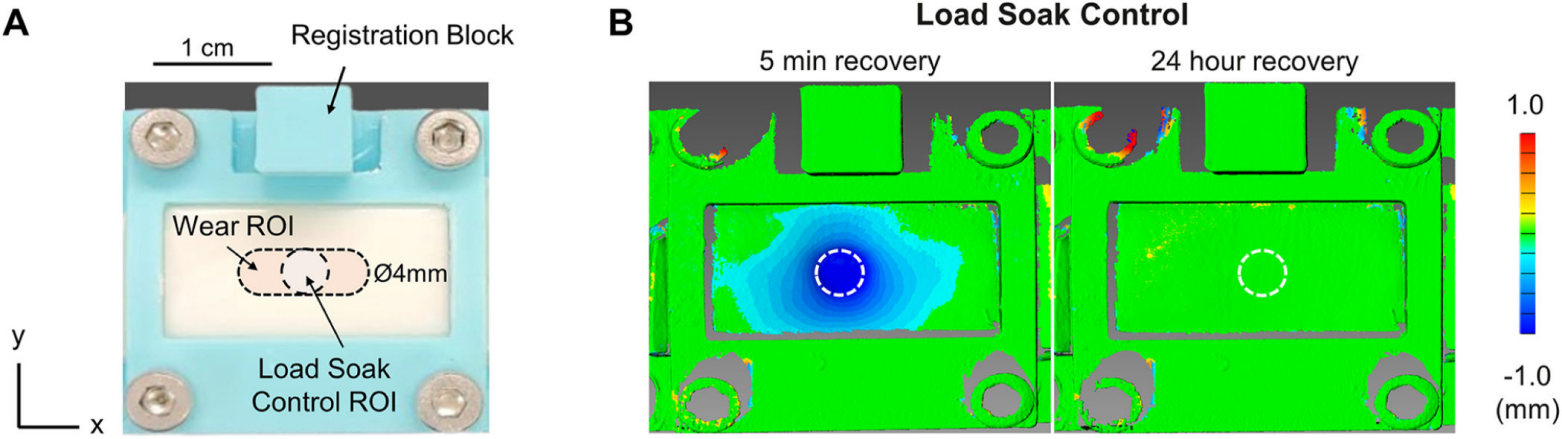
Detachable testing platform illustrating A) the designated testing ROI for both wear and load soak controls along the translational wear path (x-direction). B) 3D color maps depict the initial volume loss observed 5 min after a load soak control (1.5 MPa). Following a 24 h recovery period, the initial volume loss is fully recovered indicating that volume loss observed after wear testing is most likely due to permanent deformation, or material loss, and not viscoelastic creep.

3D color maps were generated in FlexScan3D software by registering the pre-test model to the post-recovery models using a registration block following our previously established methods (cross-sectional area = 0.53 cm^2^; accuracy = 9.7 ± 5.9 μm) (Fig. 3). (Hollar et al., 2018; Benfield et al., 2023) The testing region-of-interest (ROI) (Fig. 3A) was isolated using a defined snipping template based on the geometry of the wear path (Benfield et al., 2022, 2023). This template was identically applied to all 3D models ensuring consistency between the ROIs. Volume loss (*V*) was calculated as the linear surface deviation within the ROI between pre- and post-recovery 3D models multiplied by the area of the wear path (area = 32.6 mm^2^) or load soak control ROI (area = 12.6 mm^2^). To assess the effect of loading magnitude with the baseline unloaded controls, the volume loss of the unloaded controls was calculated within the area of the wear path. The specific wear rate *(W_s_)* was then defined as the meniscal volume loss divided by the total sliding distance (*s*) and applied load (*P*): *W*_*s*_ = *V*/(P*s) (Cilingir, 2015). Load soak controls recovered 98 ± 4% of their initial volume loss during the recovery period (Fig. 3B), indicating that the volume loss measured 24 h after wear testing was likely permanent deformation or material loss and not viscoelastic creep.

### 2.6. Dynamic mechanical analysis

To assess material weakening, we performed non-destructive dynamic mechanical analysis (DMA) on each meniscal plate before wear testing and after the 24 h recovery period using an ElectroForce 5500 mechanical test system (TA Instruments; 45N load-cell; resolution = ± 0.002 mm). With the meniscal plate clamped into the detachable platform, we applied a preload of 0.1N within the center of the testing region using an aluminum flat-ended cylindrical indenter (3.5 mm diameter; non-porous; deburred edges). The preload was followed by a preconditioning protocol of ten sinusoidal waves to 14% strain at 1.0 Hz and a ramp of 12% strain at a rate of 0.01 mm/s, followed by 10 min of stress relaxation (Yocham et al., 2018). Cyclic compression at 1% peak-to-peak amplitude was then applied for 20 cycles at 1.0 Hz (Yocham et al., 2018). All specimens were submerged in PBS during DMA testing.

Custom software (LabVIEW, v2017) was used to automatically calculate the equilibrium modulus, dynamic modulus, phase shift, and percent stress relaxation (Yocham et al., 2018). Compressive linear modulus was measured as the slope of the stress-strain curve during the 12% strain ramp. Equilibrium modulus was measured as the ratio of stress to strain at the end of the stress relaxation period. Dynamic modulus and phase shift were calculated by fitting the last four cycles of oscillation data (stress and strain) to a four-parameter sine wave function (*R*^*2*^ = 0.99 ± 0.01), where dynamic modulus was the ratio of stress amplitude to strain amplitude and phase shift was the difference between fitted phase parameters from the stress-time and strain-time data. The percent stress relaxation was measured by comparing the peak stress reached after compression to 12% strain to the stress at the end of a 10 min stress relaxation period (Yocham et al., 2018). A MATLAB script was used to calculate the relaxation time constant (τ_R_) by fitting the stress relaxation portion of the stress-time data to a three-parameter viscoelastic constitutive equation derived with a finite loading rate as described by Lin et al. (*nRMSE* = 1.1 ± 0.7%) (Lin et al., 2022). The relaxation rate was then calculated by taking the inverse of the relaxation time constant (τ_R_^−1^), where a higher relaxation rate indicates faster stress relaxation. DMA testing was done for all wear and control (load soak, unloaded) specimens to determine wear-dependent changes in mechanical compressive properties.

### 2.7. Brightfield and polarized light microscopy: staining and imaging

Half of the tested wear (*n* = 36 total), load soak control (*n* = 9 total), and unloaded control (*n* = 3 longitudinal plus an additional *n* = 3 transverse) specimens were used to evaluate fiber fraying through microscopy techniques. Therefore, a total of 51 specimens were prepped for microscopy by extracting the 4 × 11 mm ROI using a scalpel for all wear and control specimens (Fig. 3A). These subsections were then snap-frozen and stored at −80° C for at least 24 h. Using a Leica CM1950 cryostat, 25 μm thick sections were cut along the linear translational path at 1 mm intervals (Fig. 3A; x-direction) and placed on Superfrost Plus Gold Slides (Fisher Scientific, Cat #15–188-48). Three sections for each specimen were collected for a total of 153 sections. The sections were rinsed in distilled water, dipped in 10% Neutral Buffered Formalin, then incubated in Sirius Red solution prepared with saturated picric acid (Fisher Scientific, Cat #50–300-77) for 1 h at room temperature (Cui and Min, 2007). Slides were then rinsed 2x in acidified water (5 ml glacial acid to 1 L distilled water). Excess acidified water was removed and slides were dipped 3x in 100% ethanol and then washed 2x in Histoclear for 2 min each. Finally, slides were cover-slipped using Permount mounting media (Fisher Scientific, Cat#SP15–100).

Microscopy was conducted using a Leica DMi8 Microscope fitted with a CMOS Leica K3C camera for color brightfield imaging and an sCMOS Leica K8 camera for monochrome polarized light imaging. Samples were placed on a rotatable 76 × 26 mm stage and horizontally oriented with the superficial surface facing north. Images were captured with a 5x/0.15 NA objective and tiled using the Navigator module within the Leica LASX software (v3.9.1.28433). Care was taken to capture the full dynamic range (8-bit color for brightfield, 16-bit for polarized light) without saturation. Polarized light images were utilized to measure the thickness of the meniscal superficial layer following testing (see Supplemental Material), while color brightfield images were used to assess fiber fraying.

### 2.8. Grading system to assess meniscal fiber fraying

**Fig. 4.**
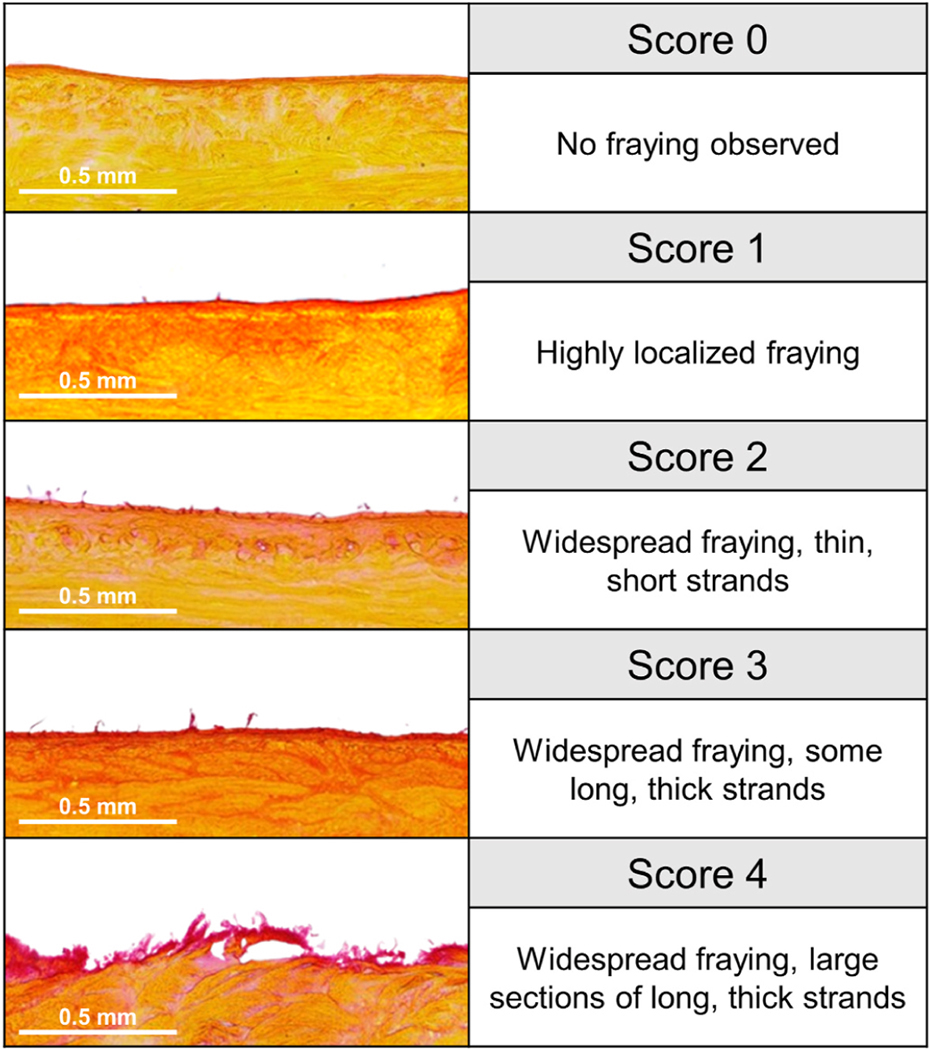
Grading system for meniscal fraying. Scores from three meniscal sections were summed to generate a total fraying score, where these cumulative scores were categorized into grades representing the level of meniscal fraying: 0–1 (grade 0), 2–4 (grade 1), 5–7 (grade 2), 8–10 (grade 3), and 11–12 (grade 4).

To our knowledge, no standardized scoring system exists to specifically quantify meniscal surface fibrillation. Therefore, we developed a custom grading system to assess the degree of fiber fraying observed in brightfield images. This system assigned scores ranging from 0 to 4 based on the presence and severity of fraying present at the superficial meniscal surface (Fig. 4). For each meniscal specimen, scores from the three sections were summed to generate a total fraying score. These cumulative scores were then categorized into grades representing the level of meniscal fraying: 0–1 (grade 0), 2–4 (grade 1), 5–7 (grade 2), 8–10 (grade 3), and 11–12 (grade 4) (Fig. 4). This classification system is adapted from established histopathological scoring of degeneration in both meniscal and articular cartilage tissues (Pauli et al., 2011; Majd et al., 2017; Pritzker et al., 2006). Scoring was performed independently by three individuals with the images randomized and test conditions blinded. Additionally, to assess intra-rater reliability, all reviewers repeated the scoring process one week after the initial evaluation. Intraclass correlation coefficients (ICCs) were used for inter- and intra-rater agreement on meniscal fraying scores (Pauli et al., 2011). Inter-rater agreement among three graders was strong, with an ICC of 0.92. Intra-rater agreement was also strong with ICC values ranging from 0.90 to 0.93.

## 2.9. Statistical analysis

Statistical software SPSS (IBM v28.0) was utilized for all statistical analysis. The effects of motion type, loading magnitude, and fiber orientation on mechanical wear behavior were assessed using three-way ANOVA. A mixed repeated-measures ANOVA was used to determine significance in pre-test and post-recovery compressive properties with the three factors listed above as between-subjects factors and time as a within-subject variable. If significance was found between-subjects, additional ANOVAs were performed using the difference data between pre-test and post-recovery compressive properties to determine significant effects within groups. Normality assumptions were checked for all ANOVA testing groups. While a small percentage of groups (~10%) deviated from normality for each dependent variable, nonparametric Kruskal-Wallis tests conducted for a subset of cases produced nearly identical p-values and trends as the ANOVAs. This is consistent with prior studies showing that ANOVA testing is a robust method for analyzing data sets with minor normality deviations (Knief and Forstmeier, 2021; Lumley et al., 2002; Schielzeth et al., 2020), thus supporting the use of ANOVAs for this study. Additionally, non-parametric Kruskal-Wallis tests and Mann-Whitney U tests were used to assess the effect of our three main factors on ranked order fraying grades. Simple and multiple regression analyses were performed to correlate compressive properties and mechanical wear behavior, excluding controls. A backwards stepwise method was used to identify predictive variables, with multicollinearity assessed via the variance inflation factor (VIF), where all final variables had VIF values below 5 (Shrestha, 2020). These regression analyses were completed in SPSS and an R based web application (Multiple Linear Regression Calculator, 2017). For all statistical tests, significance was set at *p* < 0.05 and all data are reported as mean ± standard deviation. Bonferroni adjustments were used for all pairwise comparisons. Based on our preliminary wear testing data, an a priori power analysis was performed to estimate the required sample size to detect a 25% difference in creep rate due to loading conditions with 80% power (α = 0.05), thus resulting in a target of *n* = 6 meniscal plate-cartilage pin pairs per group. To examine type II error, a posteriori 95% confidence intervals and effect sizes were calculated for mechanical wear behavior (Nakagawa and Cuthill, 2007).

## 3. Results

### 3.1. Mechanical wear behavior

#### 3.1.1. Effect of motion type

**Fig. 5.**
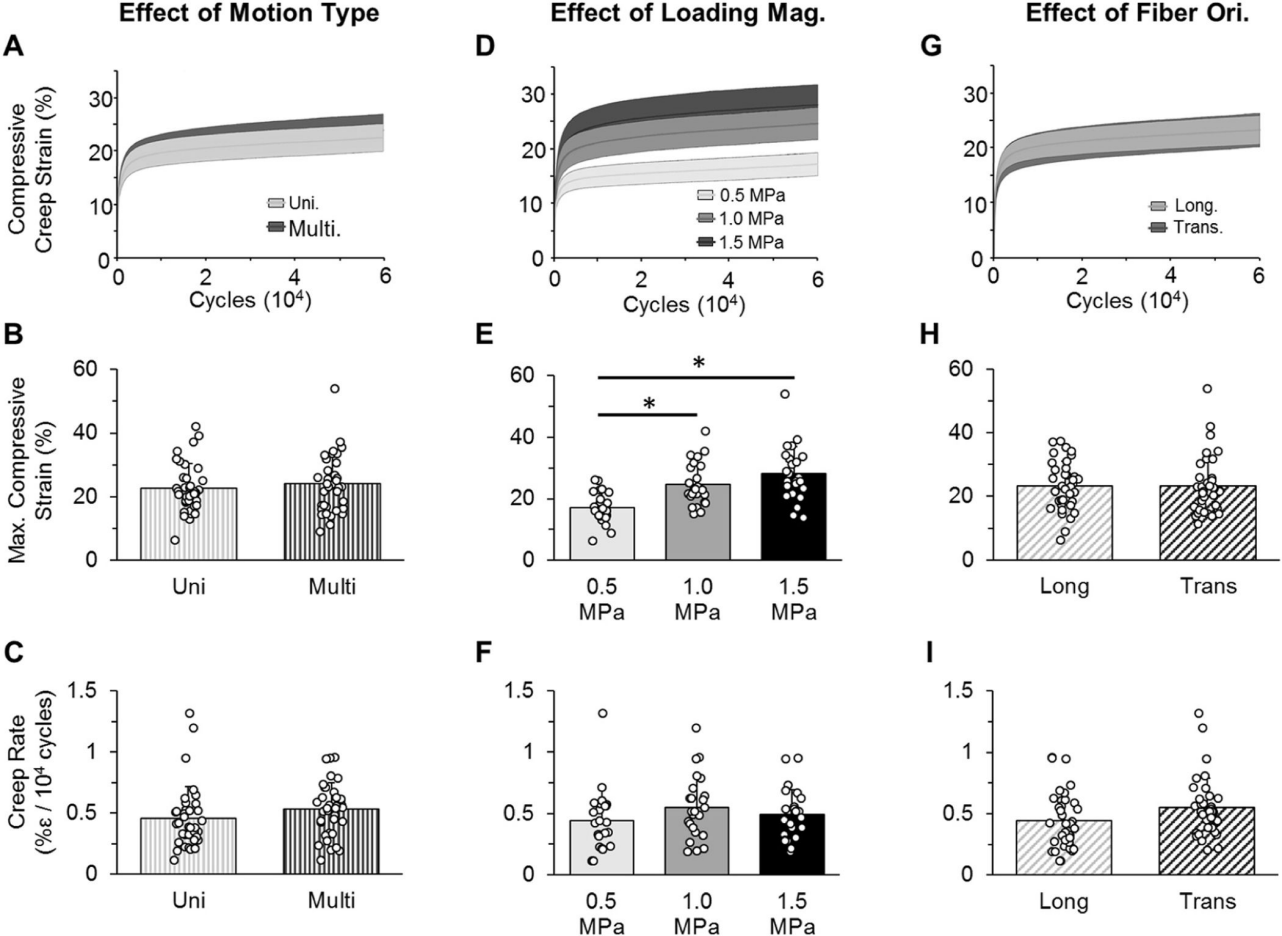
Meniscal creep behavior throughout 60K cycles of wear testing. A-C) Motion type (n = 36 per group) had no statistically significant influence on creep behavior (color bands = 95% confidence intervals), whereas D-F) increases in loading magnitude (n = 24 per group) significantly increased compressive creep strain and creep rate. G-I) The loading direction relative to fiber orientation (n = 36 per group) had no significant effect on creep behavior. * = (*p* < 0.05).

**Fig. 6.**
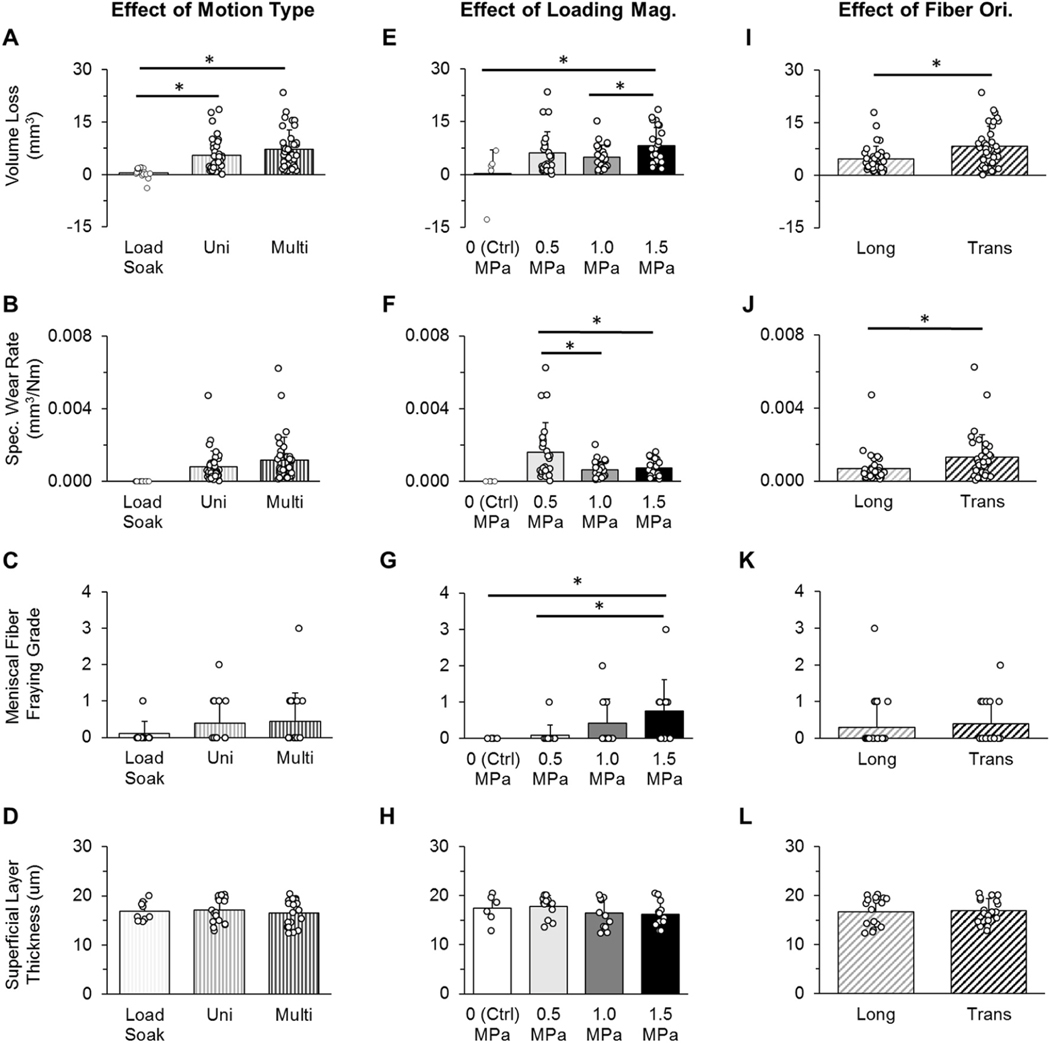
Meniscal wear behavior across motion type, loading magnitude, and fiber orientation relative to applied sliding load. **A-D)** Motion type had no statistically significant influence on meniscal wear behavior, whereas **E-H)** loading magnitude did have significant effect. Interestingly, higher loading magnitudes resulted in significantly higher volume loss and fiber fraying. **I-L)** The effect of fiber orientation relative to the applied sliding load significantly affected volume loss and specific wear, where motion applied along the transverse fiber orientation led to nearly 2x greater volume loss and specific wear rates. Sample sizes for volume loss and specific wear rates are as follows: motion type (n = 36 per group), loading magnitude (n = 24 per group), fiber orientation (n = 36 per group), load soak controls (n = 18), and unloaded controls (n = 6). For microscopy assessments of fiber fraying and superficial layer thickness, half of the tested wear, load soak control, and unloaded control specimens were used. * = (*p* < 0.05).

**Fig. 7.**
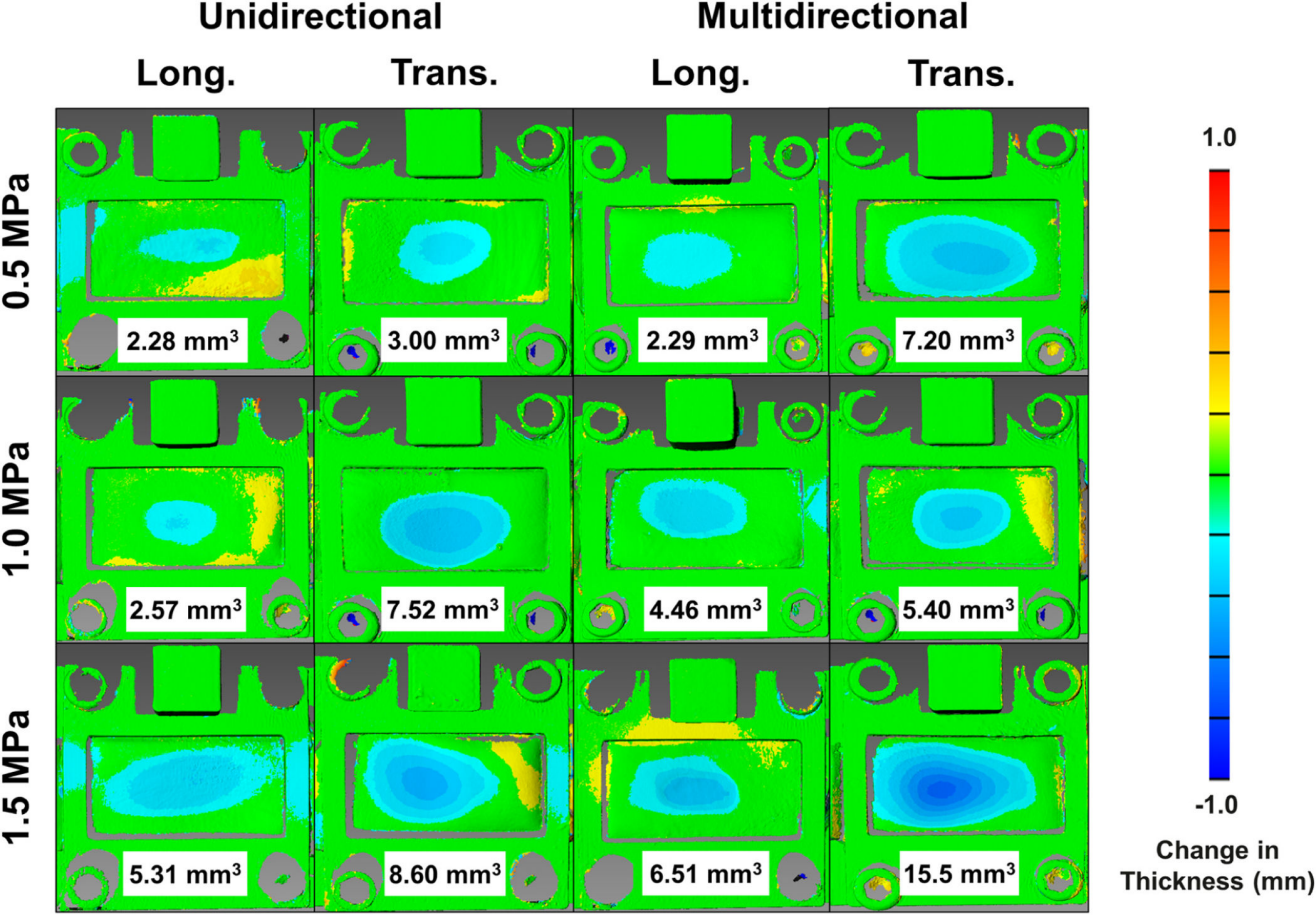
3D color maps illustrate changes in meniscal thickness (mm) after a 24 h recovery period for all wear testing groups. The volume loss of the selected specimens is representative of the average volume loss for their respective group, where differences between the displayed specimen-specific volume loss and median group volume loss are on average 0.8 ± 0.7 mm^3^.

Multidirectional motion resulted in creep rates nearly 16% higher than those under unidirectional motion, although not statistically significant (Fig. 5C). Additionally, multidirectional motion resulted in a 31% increase in meniscal volume loss (*p* = 0.13) (Figs. 6A and 7) and 42% greater specific wear rates compared to unidirectional motion (*p* = 0.13) (Fig. 6B), yet these results were likewise not significant. Load soak controls exhibited 12x and 16x less volume loss in comparison to unidirectional and multidirectional specimens, respectively (*p* < 0.001) (Fig. 6A). Furthermore, motion type did not significantly influence fiber fraying (*p* = 0.98), yet both unidirectional and multidirectional motion resulted in 3.5–4x higher fraying compared to the load soak control (*p* = 0.35, *p* = 0.35) (Fig. 6C). No significant differences in superficial layer thickness were observed between motion types (Fig. 6D).

#### 3.1.2. Effect of loading magnitude

Higher loading magnitudes had a significant large effect on compressive strain (Table 1; $\eta_{p}^{2}$ = 0.31). The 1.5 MPa loading condition increased volume loss by 36% and 70% relative to 0.5 MPa and 1.0 MPa loads, respectively (*p* = 0.33, *p* = 0.04) (Fig. 6E). This is most prominently visualized in the 3D color maps, where increased loading magnitudes show a greater shift towards darker blue hues, thus reflecting more extensive volume loss (Fig. 7). Unloaded controls exhibited 24–42x less volume loss in comparison to loaded specimens, with significant differences observed between the 1.5 MPa case (*p* = 0.006) (Fig. 6E). Interestingly, the specific wear rate for the 0.5 MPa loading condition was 2.2–2.5x greater than the 1.0 MPa and 1.5 MPa loading conditions, respectively (*p* = 0.002, *p* = 0.006) (Fig. 6F). Higher loading magnitudes increased fraying in comparison to lower loading magnitudes, with 1.5 MPa of loading having 9.4x higher fraying than the 0.5 MPa condition (*p* = 0.01) (Fig. 6G).

#### 3.1.3. Effect of fiber orientation relative to applied sliding load

The relative fiber orientation had a significant large effect on meniscal volume loss (Table 1; $\eta_{p}^{2}$ = 0.16). Compared to specimens loaded longitudinally to the circumferential fiber direction, transverse specimens had nearly a two-fold increase in volume loss compared to the longitudinal group (*p* = 0.001) (Figs. 6I and 7). Furthermore, transverse specimens resulted in a significant 87% increase in specific wear rates compared to the longitudinal specimens (*p* = 0.009) (Fig. 6J). Relative fiber orientation had no significant impact on fraying grades or on superficial layer thickness (Fig. 6K and L). Specimens loaded transversely to the circumferential fiber direction resulted in creep rates nearly 24% higher than those loaded longitudinally, although not statistically significant (Fig. 5I)

### 3.2. Meniscal compressive properties

#### 3.2.1. Initial compressive properties

Average initial compressive properties of all specimens (wear, load soak controls, unloaded controls) were as follows: equilibrium modulus, 0.54 ± 0.34 MPa; dynamic modulus, 2.68 ± 1.80 MPa; linear modulus, 1.90 ± 1.46 MPa; stress relaxation, 50.1 ± 9.9 %; relaxation rate, 0.008 ± 0.008 s^−1^, and phase shift, 0.14 ± 0.04 rad. No significant differences in initial compressive properties were detected between motion type, loading magnitude, or fiber orientation groups. A comprehensive table for all pre- and post-testing compressive properties can be found in the Supplemental Material.

#### 3.2.2. Effect of motion type

**Fig. 8.**
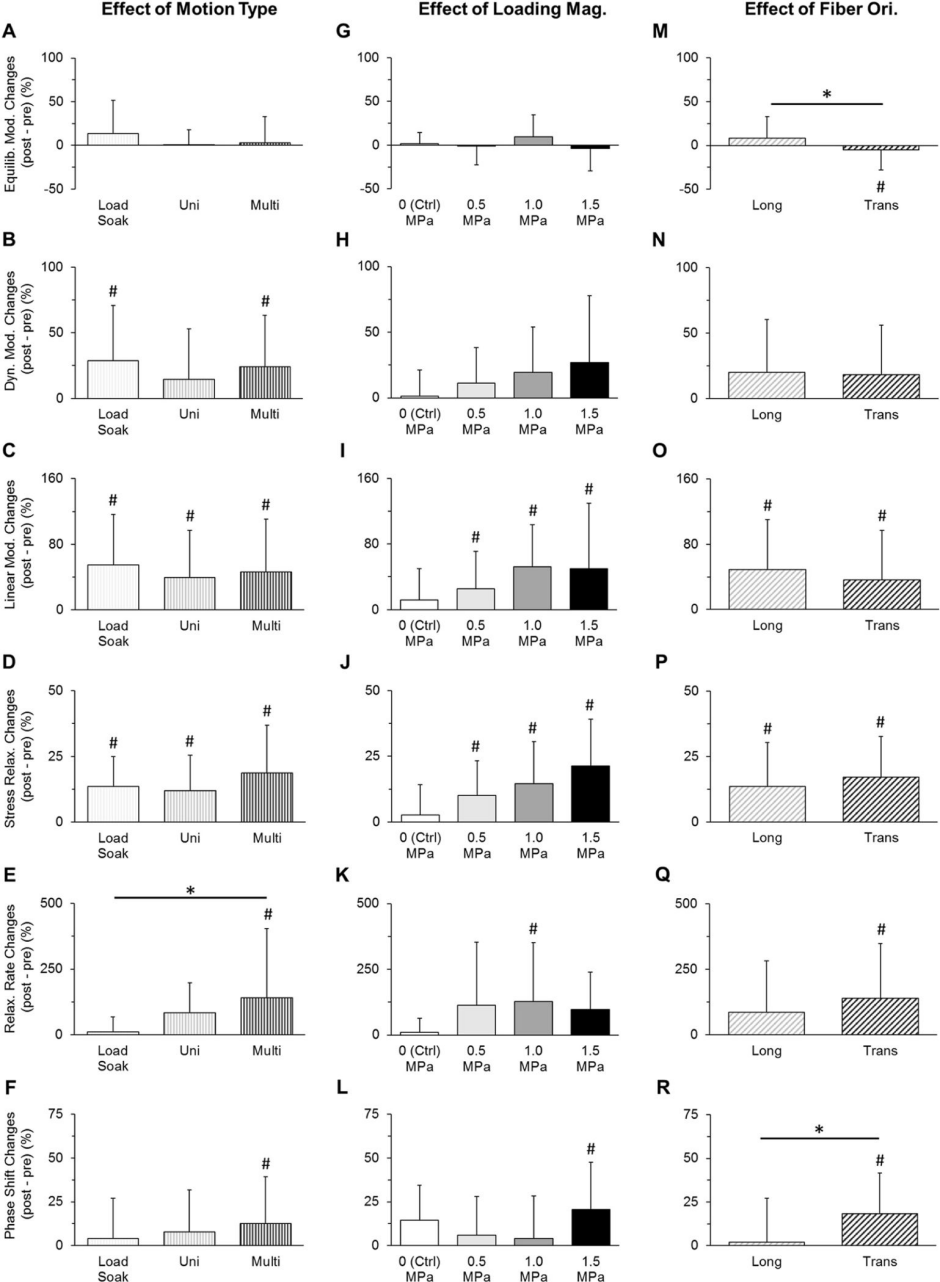
Effects of motion type, loading magnitude, and fiber orientation on changes in compressive properties before and after wear testing. Changes were calculated by subtracting pre-test compressive properties from post-test values (post – pre). **A-F)** Motion type (n = 36 per wear group; n = 18 per load soak) had no significant effect on changes in compressive properties following testing. **G-L)** Loading magnitude (n = 24 per wear group; n = 6 per unloaded control) also did not have a significant effect, however, dynamic modulus, linear modulus, and stress relaxation increased with increasing compressive magnitudes. **M-R)** Fiber orientation (n = 36 per group) significantly affected the phase shift where sliding load applied along the transverse fiber orientation resulted in greater differences in phase shift following testing. # = significant difference from pre-to post-testing within-group. * = significant difference between-groups (*p* < 0.05).

Dynamic mechanical analysis revealed that all compressive properties significantly increased under multidirectional motion from pre-to-post testing, with the exception of equilibrium modulus (Fig. 8A–F). Significant pre-to-post increases in linear modulus and stress relaxation were also observed for both load soak and unidirectional specimens (Fig. 8C and D). Minimal percent changes were seen in equilibrium modulus between motion types and load soak control, however, load soak controls had nearly 5x greater percent changes in equilibrium modulus in comparison to the multidirectional specimens, although not significant (Fig. 8A). Moreover, multidirectional motion had 1.7x and 12.5x greater percent changes in relaxation rate in comparison to unidirectional (*p* = 0.58) and load soak specimens (*p* = 0.049), respectively (Fig. 8E). No significant differences were detected between motion types for any percent change in compressive properties.

#### 3.2.3. Effect of loading magnitude

All loaded specimens exhibited significant increases in stress relaxation from pre-to post-testing (Fig. 8J). While not statistically significant, percent changes in dynamic modulus, linear modulus, and stress relaxation all exhibited increasing trends with greater loading magnitudes, where values at 1.5 MPa were approximately twice those at 0.5 MPa (Fig. 8H–J). Phase shift percent changes at 1.5 MPa were 3.4–5.1x greater than at the 0.5 MPa and 1.0 MPa magnitudes, respectively, though these differences were also not significant (Fig. 8L). Overall, no significant differences were found between loading magnitudes for any percentage change in compressive properties (Fig. 8G–L). Additionally, no significant differences were detected for the unloaded controls pre-to post-testing for any of the evaluated compressive properties.

#### 3.2.4. Effect of fiber orientation relative to applied sliding load

All compressive properties significantly increased or decreased when sliding load was applied relative to the transverse fiber orientation from pre-to-post testing, with the exception of dynamic modulus (Fig. 8M–R). Interestingly, equilibrium modulus increased from pre-to post-testing in longitudinal specimens but significantly decreased in transverse specimens, with a significant difference in percentage changes between the two groups (*p* = 0.02) (Fig. 8M). Compared to specimens loaded longitudinally to the circumferential fiber direction, transverse specimens resulted in 63% faster relaxation, although not significant (*p* = 0.26) (Fig. 8Q). Moreover, transverse specimens experienced nearly 9x greater percent changes in phase shift compared to longitudinal specimens (*p* = 0.01) (Fig. 8R).

### 3.3. Correlations and interactions

#### 3.3.1. Relationship between meniscal thickness, cartilage height loss, and mechanical wear behavior

No significant differences in initial meniscal thickness and cartilage pin-height loss were observed within groups for motion type, loading magnitude, and fiber orientation. However, initial meniscal thickness demonstrated a correlation with maximum compressive strain, where thicker specimens were associated with less maximum compressive strain (*p* = 0.004, *r* = −0.29). No other significant relationships were identified between meniscal thickness, pin-height loss, and other wear behaviors.

#### 3.3.2. Relationship between mechanical wear behavior and initial compressive properties

**Fig. 9.**
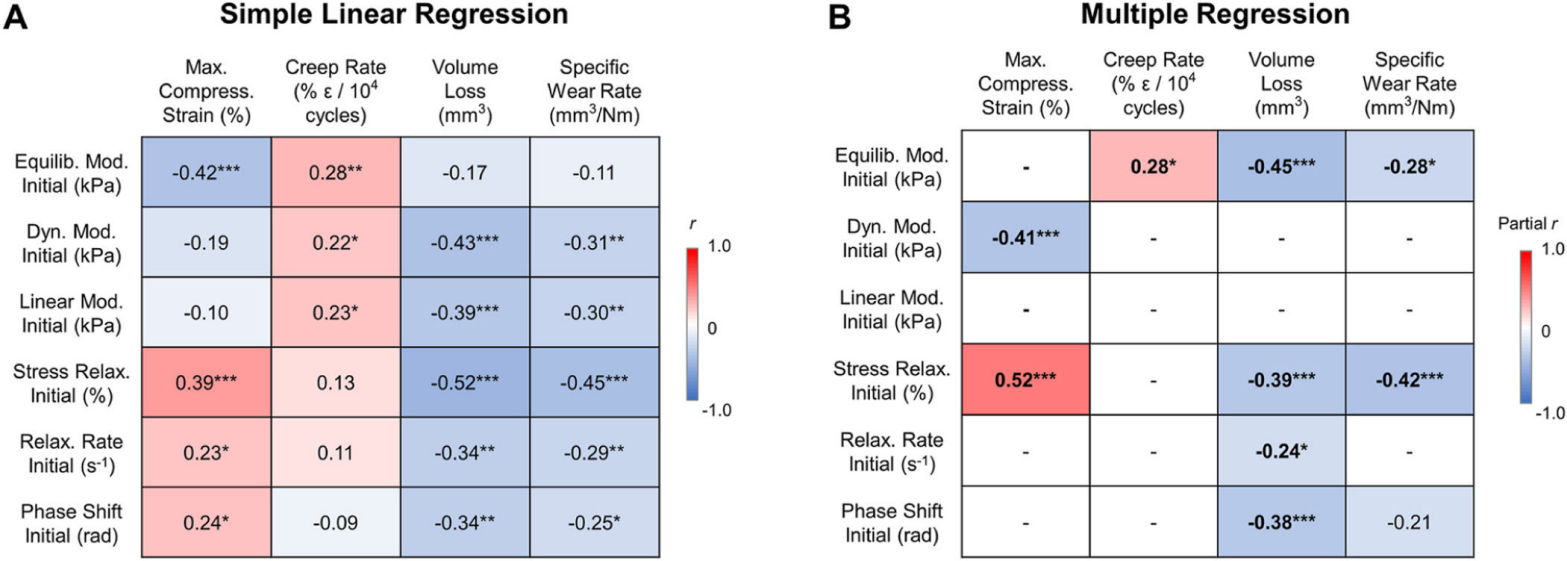
Initial compressive properties can predict meniscal wear behavior. Values are correlation coefficients from A) simple linear regression (*r*) and B) the final iteration of multiple regression with backwards stepwise analysis (partial *r*). Sample size *n* = 72 per cell. Bold = Identified as a significant predictor of wear behavior from backward stepwise method. Empty cell = excluded variable from backward stepwise method. * = (*p* < 0.05), ** = (*p* < 0.01), *** = (*p* < 0.001).

Simple regression analysis revealed significant correlations between initial compressive properties and wear behavior (Fig. 9A). Notably, initial stress relaxation showed significant moderate correlations for maximum compressive strain (*p* = 0.001, *r* = 0.39), volume loss (*p* = 0.001, *r* = −0.52), and specific wear rate (*p* = 0.001, *r* = −0.45). Comparable significant correlations were also identified for both the initial relaxation rate and phase shift across these mechanical outcomes (Fig. 9A). Interestingly, initial equilibrium modulus exhibited a significant negative correlation with maximum compressive strain (*p* = 0.001, *r* = −0.42) and a significant positive correlation with creep rate (*p* = 0.009, *r* = 0.28). Moreover, initial dynamic and linear moduli showed significant weak-moderate correlations for creep rate, volume loss, and specific wear rate (Fig. 9A).

Multiple regression indicated a moderate collective significant effect between initial compressive properties and maximum compressive strain ($R_{\mathrm{adj}}^{2}$ = 0.27). Using a backward stepwise approach, initial dynamic modulus (*p* = 0.001, partial *r* = −0.41) and initial stress relaxation (*p* = 0.001, partial *r* = 0.52) were identified as significant predictors of maximum compressive strain, while initial equilibrium modulus was identified as a significant predictor of creep rate (*p* = 0.02, partial *r* = 0.28) (Fig. 9B). Additionally, multiple regression analysis indicated a strong collective significant effect of initial compressive properties on volume loss (*p* < 0.001, $R_{\mathrm{adj}}^{2}$ = 0.42) where equilibrium modulus (*p* = 0.001, partial *r* = −0.45), stress relaxation (*p* = 0.001, partial *r* = −0.39), relaxation rate (*p* = 0.045, partial *r* = −0.24), and phase shift (*p* = 0.001, partial *r* = −0.38) were identified as significant predictors (Fig. 9B). Similarly, a moderate collective significant effect existed between initial compressive properties and specific wear (*p* = 0.001, $R_{\mathrm{adj}}^{2}$ = 0.19), with equilibrium modulus (*p* = 0.02, partial *r* = −0.28), stress relaxation (*p* = 0.001, partial *r* = −0.42), and phase shift (*p* = 0.08, partial *r* = −0.21) identified as predictors (Fig. 9B).

#### 3.3.3. Interactions between wear testing factors

No statistically significant interactions were detected among the three main factors for meniscal wear behavior: motion type*loading magnitude (*p* = 0.09), motion type*fiber orientation (*p* = 0.69), loading magnitude*fiber orientation (*p* = 0.07), or the three-way interaction motion type*loading magnitude*fiber orientation (*p* = 0.66). Meniscus type (medial vs. lateral) had no significant effect on any of our wear behavior (*p* = 0.24), while meniscus region (anterior v. posterior) did have an effect on compressive strain with posterior regions having higher maximum compressive strain than anterior regions (*p* = 0.03; effect size: $\eta_{p}^{2}$ = 0.07). No significant interaction was found between meniscus and region (*p* = 0.14), nor were there significant interactions between meniscus type, region, and our main factors.

## 4. Discussion

Given the high prevalence of meniscal degeneration, it is imperative to identify key biomechanical factors contributing to this insidious disease. The goal of this study was to evaluate the effects of various contact conditions (i.e. motion type and loading magnitude) on anisotropic meniscal wear behavior. To the best of our knowledge, this is the first study to demonstrate that the meniscus exhibits anisotropic wear behavior that results in volume loss, fraying, and alterations in mechanical properties. Our findings underscore the importance of joint loading environments on meniscal health and degeneration.

A key finding from this study is that the magnitude of applied loading strongly influences meniscal wear behavior, with increases in loading magnitude leading to greater compressive strain, volume loss, and surface fraying (Fig. 5D–E, Fig. 6E–G). Maximum compressive strain increased with higher applied loads, most likely due to the viscoelastic creep response of the tissue during prolonged wear testing (Fig. 5E). More notably, the highest loading condition (1.5 MPa) significantly increased volume loss by 36% and 70% relative to 0.5 MPa and 1.0 MPa loads, respectively (Fig. 6E). Meniscal fiber fraying also increased in a load dependent manner. The highest loading condition resulted in 9.4x more fraying compared to the lowest load (Fig. 6G). This trend in fraying aligns with previous findings in a bovine cartilage wear study by Petersen et al. (2023) that found repetitive compressive loading caused surface delamination and matrix disruption (Petersen et al., 2023). These results therefore suggest that prolonged exposure to higher magnitudes of joint loading (i.e. body weight, body-borne loads, daily activity) may be detrimental to meniscal integrity. This is further supported by a histopathological study of human meniscus, where Wesdorp et al. found a positive correlation between body mass index (BMI) and the degree of human meniscal degeneration (Wesdorp et al., 2020), thus indicating increased body mass may predispose the meniscus to degenerative changes. Interestingly, we found the specific wear rate was lowest at the highest loading magnitude (Fig. 6F). This suggests that meniscal tissue exhibits increased wear resistance at higher loads. One possible explanation for this is the formation of a protective boundary lubrication layer on the tissue surface under greater compressive stress, thus reducing friction during articulation (Petersen et al., 2023; Ghosh et al., 2014).

Motion type had less of an impact on meniscal wear behavior than we hypothesized. We observed no statistically significant differences in mechanical wear behavior, fraying, or compressive property changes between unidirectional and multidirectional motion paths. This result goes against our hypothesis, and is in contrast to previous polyethylene wear studies (Bragdon et al., 1996; Kang et al., 2008), and to a prior cartilage-on-cartilage frictional study which found approximately 60% increases in cartilage surface roughness for multidirectional motion in comparison to unidirectional (Northwood and Fisher, 2007). Furthermore, a study by Cilingir et al. showed that rotation-only motion resulted in 44x greater specific wear rates compared to simple sliding motion in bovine articular cartilage (0.04 mm^3^/Nm vs 0.001 mm^3^/Nm, respectively) (Cilingir, 2015). In contrast, we saw no significant difference between unidirectional and multidirectional specific wear rates (Fig. 6B), though the Cilingir et al. test ran for nearly 3x more cycles than ours. It is important to note that even though we detected no significant differences due to motion type, multidirectional motion did have 31% higher volume loss and 42% greater specific wear rates compared to unidirectional motion (Fig. 6A and B). This suggests that the cross-shear phenomena observed in polyethylene may still influence wear in meniscal tissue, and raises the possibility that prolonged or repetitive activities associated with multidirectional motion (e.g., stair climbing, pivoting) may have long-term effects contributing to cumulative meniscal wear.

Surprisingly, the orientation of the circumferential collagen fibers relative to the applied sliding load was an important factor in predicting wear behavior. Since wear testing was performed on the superficial layer, with a randomly oriented collagen network, we had expected to see no differences in wear behavior between fiber groups. However, the transverse specimens experienced 1.8x greater volume loss and 1.9x higher specific wear rates than the longitudinal fiber specimens (Fig. 6I and J). Mechanical wear behavior of meniscus is therefore anisotropic, and the underlying circumferential alignment of the inner meniscal fibrous layers has an influence on mechanical wear resistance. This is comparable to a study by Hossain et al. that determined articular cartilage wear to be anisotropic with 2.2x greater amounts of glycos-aminoglycans (GAGs) released from the cartilage during transverse wear than longitudinal wear (Hossain et al., 2020). Interestingly for our study, the higher volume loss for the transverse specimens, in combination with minimal fiber fraying (Fig. 6K) and minimal superficial thinning (Fig. 6L), suggests that greater damage occurred within the inner and deep tissue layers. In recent studies using polarized Raman spectroscopy and micro CT, a loss of circumferential fiber orientation was pinpointed as a defining trait of degenerative meniscus, underlining the critical role of fiber alignment in maintaining the structural integrity of meniscal tissue (Karjalainen et al., 2021; Prokopi et al., 2021). Therefore, our results highlight the importance of considering fiber orientation relative to loading direction in future mechanical wear experiments and computational wear models.

Dynamic mechanical analysis revealed the influence of wear testing on tissue compressive properties (Fig. 8). In particular, multidirectional motion resulted in 1.6–1.7x greater percent changes in stress relaxation, relaxation rate, and phase shift (all indicators of energy dissipation) compared to unidirectional motion, though these differences were not significant (Fig. 8D–F). These trends may point to subtle matrix disruption under more complex loading paths where continuous cross-shear conditions alter viscous dampening. Furthermore, we observed a trend of increasing changes in linear and dynamic moduli with increases in loading magnitude (Fig. 8H and I), most likely due to matrix compaction. This stiffening response is consistent with previous findings from Seitz et al. which found that in OA development, stiffening of the meniscal surface was detected as a first sign of OA-related knee degeneration (Seitz et al., 2021). Most notably, specimens loaded transversely to the circumferential fiber direction exhibited a significant decrease in equilibrium modulus from pre-to post-testing, yet showed a significant increase in stress relaxation and a non-significant increase in dynamic modulus (Fig. 8M–N, P). This pattern suggests matrix weakening accompanied by increased fluid exudation, thus requiring greater dynamic stress to achieve comparable dynamic strain. As previously noted, despite greater volume loss, the transverse specimens had minimal changes in fiber fraying and superficial layer thinning (Fig. 6K and L). The observed changes in compressive properties therefore further support the idea that damage occurred deeper within the matrix rather than at the superficial layer. Of note, unloaded controls did not show significant changes in compressive properties during the roughly 33 h total test duration (Fig. 8G–L), but load soak controls did have an increase in moduli from pre-to post-testing (Fig. 8A–C). This may reflect ongoing collagen realignment in the absence of motion (Sizeland et al., 2020).

**Fig. 10.**
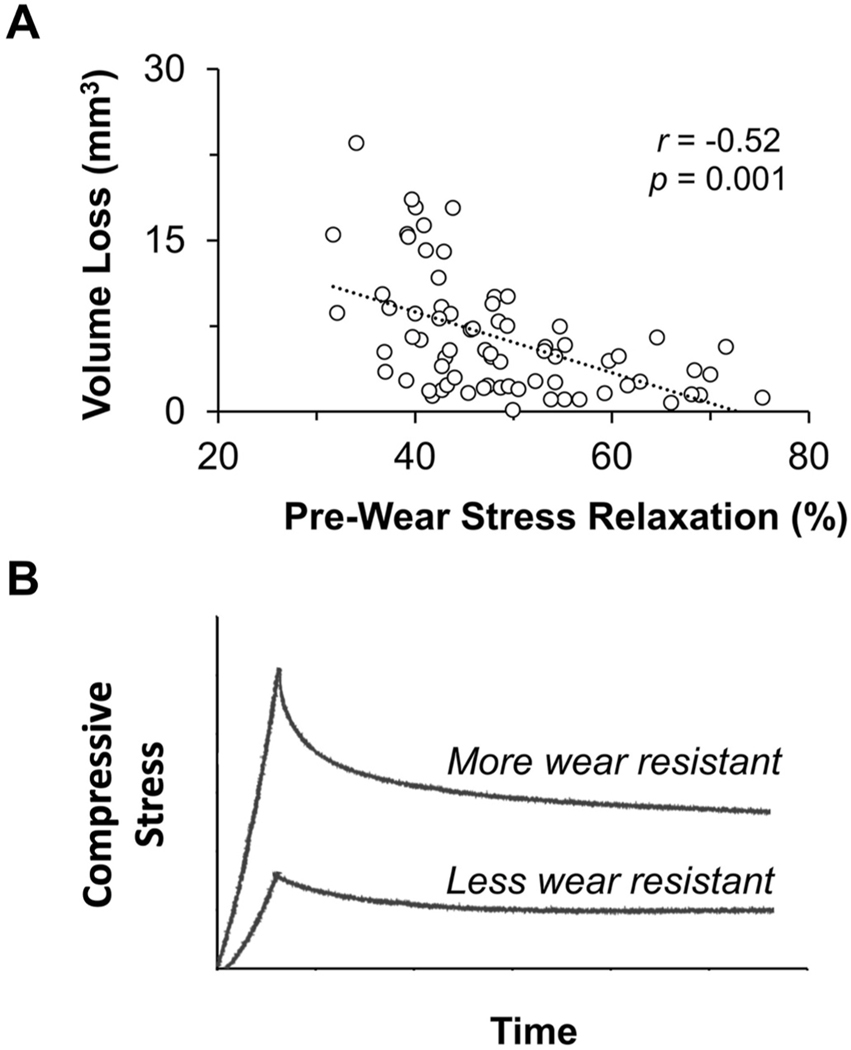
Baseline mechanical properties predict wear. A) Greater initial stress relaxation (prior to wear testing) correlates with lower meniscal volume loss during wear testing. B) Illustration of stress relaxation curves for two specimens at equivalent constant strain. Our findings suggest that meniscal tissue with greater stiffness and greater energy dissipation will be more resistant to wear.

This study also identified mechanical determinants of wear sensitivity. We found that initial moduli and stress relaxation were both significant predictors of maximum compressive strain, volume loss, and specific wear during testing (Fig. 9B). For example, specimens with initially higher equilibrium modulus and stress relaxation experienced significantly less volume loss during wear testing (Figs. 9B and 10A). This relationship can be visualized with stress–time relaxation curves at constant strain; tissues with greater initial stiffness combined with effective energy dissipation may have an increased resistance to mechanical wear (Fig. 10B). This suggests that targeting both equilibrium stiffness and relaxation in tissue-engineered or synthetic meniscus replacements may help improve wear durability, and these compressive properties may potentially serve as biomarkers for wear susceptibility. For example, non-invasive clinical imaging techniques, such as elastography and quantitative magnetic resonance imaging, could potentially assess the meniscal stiffness and viscoelastic behavior (Arunachalam et al., 2017; Rauscher et al., 2008) of meniscal tissue to determine susceptibility to wear-and-tear.

While direct studies on meniscal wear behavior are limited, our findings can be benchmarked through prior tribological and mechanical testing of cartilage and meniscus. Lizhang et al. found that linear wear in bovine cartilage increased with contact stress, with severe damage above 8 MPa (Lizhang et al., 2011). Although we used lower loads, we also saw a trend toward more fibrillation at higher loading magnitudes (Fig. 6G). Additionally, Majd et al. performed unidirectional wear testing between bovine meniscus-cartilage interfaces and found that the COF at 0.4 MPa was more than twice that at 1.0 MPa (Majd et al., 2017). This can be attributed to greater interstitial fluid pressurization and weeping at higher loads (Majd et al., 2017; Ateshian, 2009) which formed a thicker fluid film at the interface. These findings further support that a similar fluid-driven mechanism may have contributed to the lower specific wear rates we observed in meniscus at higher loads (Fig. 6F). Our anisotropic meniscal wear findings can be corroborated by Wang et al. who conducted pin-on-plate tests with bovine cartilage against metal and found that sliding motion applied transverse to the principle fiber orientation caused approximately 60% greater wear depths than longitudinal loading (Wang et al., 2025). Moreover, Abraham et al. reported that bovine meniscus exhibits significantly lower shear modulus when loaded transversely versus longitudinally (Abraham et al., 2011), which may help explain the greater volume loss we observed in transverse specimens (Fig. 6I). The initial compressive properties found in this study can also be benchmarked with previously published values for bovine meniscus. Our initial equilibrium modulus (0.54 ± 0.34 MPa) is similar to the reported range of 0.11–0.51 MPa in bovine meniscus (Baro et al., 2012; Sweigart et al., 2004), and the initial linear modulus (1.9 ± 1.5 MPa) is comparable to the 1.34 MPa reported by Coluccino et al. in bovine meniscus under similar unconfined compression conditions (Coluccino et al., 2017). Likewise, our measured initial phase shift (0.14 ± 0.04 rad) is similar to the 0.16–0.22 rad range reported for bovine meniscal tissue (Coluccino et al., 2017; Murphy et al., 2019). Our initial stress relaxation (50 ± 9.9%) was lower than the 89% reported by Coluccino et al., perhaps potentially due to the shorter relaxation period used in our protocol (600s vs. 1800s) (Coluccino et al., 2017).

This study used bovine meniscal tissue as a surrogate for human tissue, and therefore, species-specific differences in mechanical properties should be considered. Bovine menisci generally exhibit greater stiffness than human tissue, with equilibrium moduli (0.11–0.51 MPa) (Baro et al., 2012; Sweigart et al., 2004) exceeding the 0.02–0.23 MPa range reported for humans (Warnecke et al., 2017; Chia and Hull, 2008; Danso et al., 2015; Nordberg et al., 2016; Schwer et al., 2024). However, aggregate modulus ranges overlap between bovine (0.12–0.41 MPa) (Proctor et al., 1989; Sweigart et al., 2004; Danso et al., 2018; Joshi et al., 1995) and human tissue (0.10–0.40 MPa) (Sweigart et al., 2004; Schwer et al., 2024; Joshi et al., 1995). Moreover, similar phase shift values have been reported between species (bovine: 0.16–0.22 rad; human: 0.12–0.18 rad) (Murphy et al., 2019; Danso et al., 2015; Pereira et al., 2014), suggesting comparable viscoelastic behavior. Bovine meniscus therefore provides a reasonable surrogate for human tissue, though species differences in stiffness may influence wear behavior and our results should be interpreted accordingly.

Our in vitro findings can be considered in the context of in vivo meniscal degeneration. Prior studies have reported decreases in the equilibrium modulus of human meniscus with increasing degeneration and osteoarthritis severity (Warnecke et al., 2020; Fischenich et al., 2015). We observed a similar trend of reduced equilibrium modulus for the 1.5 MPa loading condition and the transverse specimens (Fig. 8G and M). Additionally, Warnecke et al. reported that with an increasing degree of degeneration the water content and the hydraulic permeability of human menisci significantly increases (Warnecke et al., 2020). These findings align conceptually with our observed increases in phase shift post-testing and reflect greater fluid mobility contributing to viscous damping (Fig. 8F, L, 8R). Furthermore, meniscal surface fraying is a hallmark of meniscal degeneration (Pauli et al., 2011), and we found that fraying increased with loading magnitude (Fig. 6G). While species differences limit direct comparisons, these studies help contextualize our findings and suggest that the mechanical changes we observed are consistent with early degenerative changes.

A unique aspect of this study is the utilization of 3D scanning to accurately quantify and visualize meniscal volume loss (Fig. 7). 3D color maps were generated by registering pre-test 3D models to the post-recovery 3D models using a registration block (Fig. 3A). Based on our previous 3D model registration study (Benfield et al., 2023), four orthogonal block surfaces were used for feature matching registration (total surface area = 2.1 cm^2^; 8.7% scanning FOV) resulting in excellent registration accuracy (9.7 ± 5.9 μm). Using our 3D optical scanning method, we detected mean volume losses of 5.6 mm^3^ and 7.3 mm^3^ for unidirectional and multidirectional motion, respectively. These volume losses represent a 6–9% change in the total meniscal volume under the wear path (Fig. 2A). By examining the 95% confidence intervals (Table 1), we can then state with 95% confidence that relative to unidirectional motion, multidirectional motion did not cause total meniscal volume to be reduced by more than 5%. Similarly, we can state with 95% confidence that high magnitude loads and fiber orientation did not cause total meniscal volume to be reduced by more than 6% and 7%, respectively. By enabling precise visualization and quantification of meniscal volume loss through 3D modeling and colorimetric mapping, this method expands upon traditional biotribological metrics. For example, COF is the gold standard in wear testing of biological materials, where implicitly, a high COF value has been assumed to produce high wear. Yet, Caligaris et al. showed that advancing osteoarthritic degeneration does not increase the friction coefficient of human cartilage (Caligaris et al., 2009), suggesting that OA related wear may progress without a concomitant increase in friction (Petersen et al., 2023; Durney et al., 2020). Unlike COF, volume loss is a direct measure of mechanical wear behavior, and therefore can provide valuable insight into factors that influence joint degeneration and OA.

This study does have limitations. First, we used PBS as a baseline lubricant for all our experiments, and compared to synovial fluid, PBS has a much lower viscosity and lacks essential macromolecules that provide boundary lubrication. This likely led to higher wear rates, but nevertheless, the use of PBS allowed us to consistently compare the effects of different loading conditions on meniscal wear behavior. Second, this study focused on the volume loss aspect of mechanical wear and did not consider material losses and associated biochemical changes. Future studies could quantify wear debris in terms of GAG and collagen loss in the lubricant to link mechanical wear to biochemical degradation (Hossain et al., 2020; Majd et al., 2017). Third, we did not directly measure the meniscal surface roughness prior to testing. However, histological evaluation of the load soak control group revealed smooth superficial layers (Fig. S6), thus indicating that initial surface roughness was likely consistent across specimens. Fourth, wear testing was performed at a single frequency (2 Hz), and wear resistance is likely dependent on the loading frequency (higher frequencies = more wear resistance). Future work could therefore evaluate how variations in loading frequency influence meniscal wear behavior. Lastly, the focus of this study was on meniscal wear and we did not consider damage or wear occurring to the cartilage. We did, however, verify that meniscal thickness loss was 3.4x greater than cartilage height loss (Supplemental Material), and therefore the cyclic creep results (Fig. 5) are predominately indicating changes in thickness of the meniscus, not the cartilage.

## 5. Conclusion

In summary, we conducted accelerated wear tests using custom pin- on-plate systems to characterize meniscal wear under various contact conditions (i.e. motion type, loading magnitude, fiber orientation). Key takeaways from this study are that loading magnitude and fiber orientation are factors that drive meniscal mechanical wear behavior, and baseline mechanical properties (such as stress relaxation and stiffness) may serve as mechanical biomarkers of meniscal wear resistance. Our findings provide evidence of a direct mechanical pathway for meniscal degeneration that can occur in the absence of biochemical alterations. Furthermore, this study offers mechanistic insights that may help advance preventative care, functional tissue engineering, and therapeutic interventions.

## Supplementary Material

Lujan Supplementary data

## Figures and Tables

**Table 1:** Comparison of mechanical wear behavior between different motion types, loading magnitudes, and fiber orientations.

	Motion Type				Loading Magnitude				Fiber Orientation			
	Uni. (*n* = 36)	Multi. (*n* = 36)	Mean Difference 95% CI	Effect Size ($\eta_{p}^{2}$)	0.5 MPa (*n* = 24)	1.5 MPa (*n* = 24)	Mean Difference 95% CI	Effect Size ($\eta_{p}^{2}$)	Long. (*n* = 36)	Trans. (*n* = 36)	Mean Difference 95% CI	Effect Size ($\eta_{p}^{2}$)

Max Compress. Strain (%)	22.6 ± 7.7	24.0 ± 9.0	[−5.3, 2.5]	0.01	17.2 ± 5.0	28.1 ± 8.8	[−15.5, −6.3]	**0.31** ^*^	23.3 ± 7.8	23.3 ± 9.1	[−3.8, 4.0]	0.001
Creep Rate (%ε/10^4^ cycles)	0.46 ± 0.26	0.53 ± 0.22	[−0.18, 0.04]	0.03	0.44 ± 0.25	0.49 ± 0.21	[−0.18, 0.09]	0.04	0.44 ± 0.23	0.55 ± 0.24	[−0.22, 0.01]	0.06
Volume Loss (mm^3^)	5.6 ± 4.6	7.3 ± 5.6	[−4.1, 0.73]	0.04	6.1 ± 6.1	8.3 ± 5.1	[−5.4, 1.0]	0.10	4.6 ± 3.7	8.2 ± 5.8	[−6.0, −1.3]	**0.16** *
Specific Wear Rate (mm^3^/Nm)	8.2E-4 ± 8.5E-4	1.2E-3 ± 1.3E-3	[−7.3E-4, 2.4E-4]	0.02	1.6E-3 ± 1.6E-3	7.3E-4 ± 4.5E-4	[5.9E-5, 1.4E-3]	**0.14** *	6.9E-4 ± 7.9E-4	1.3E-3 ± 1.3E-3	[−9.8E-4, −1.9E-5]	**0.07** *

* Effect sizes in bold represent a significant difference (*p* < 0.05), where $\eta_{p}^{2}$ = 0.01 is a small effect, $\eta_{p}^{2}$ = 0.06 is a medium effect, and $\eta_{p}^{2}$ = 0.14 is a large effect.

## Data Availability

Data will be made available on request.

## Appendix A. Supplementary data

Supplementary data to this article can be found online at https://doi.org/10.1016/j.jmbbm.2025.107212.
