# Supplementary material for: Anisotropic wear behavior of meniscus: Influence of cross-shear and loading magnitude
Source: J Mech Behav Biomed Mater. Author manuscript; Available in PMC 2026 Jul 9. (PMC13349344; doi:10.1016/j.jmbbm.2025.107212)
Supplement: Lujan Supplementary data [file NIHMS2173250-supplement-Lujan_Supplementary_data.docx]

# SUPPLEMENTAL MATERIAL

## Meniscal Flattening

To achieve a level surface for the meniscal plate, we developed a methodology to flatten the

natural curvature of the meniscus. The meniscus was first sectioned into anterior and posterior wedges. Each wedge was then compressed between two acrylic plates of a flattening device **(Fig. S1)**. The device and meniscus wedge were frozen at -4C for 24h to ensure that the tissue remained flat. Following our previously established methods, the raw flatten meniscus was then packed with cellu-clay and layered using a commercial deli slicer.^1^ The end process resulted in a flat meniscal plate ready for testing. Non-destructive indention tests were performed on 4-mm diameter meniscal plugs from both flattened and unflattened tissue using dynamic mechanical analysis. Minor differences were found in compressive properties between flattened and unflattened tissue (*p* > 0.48; *n* = 6 per group) **(Fig. S2)**.


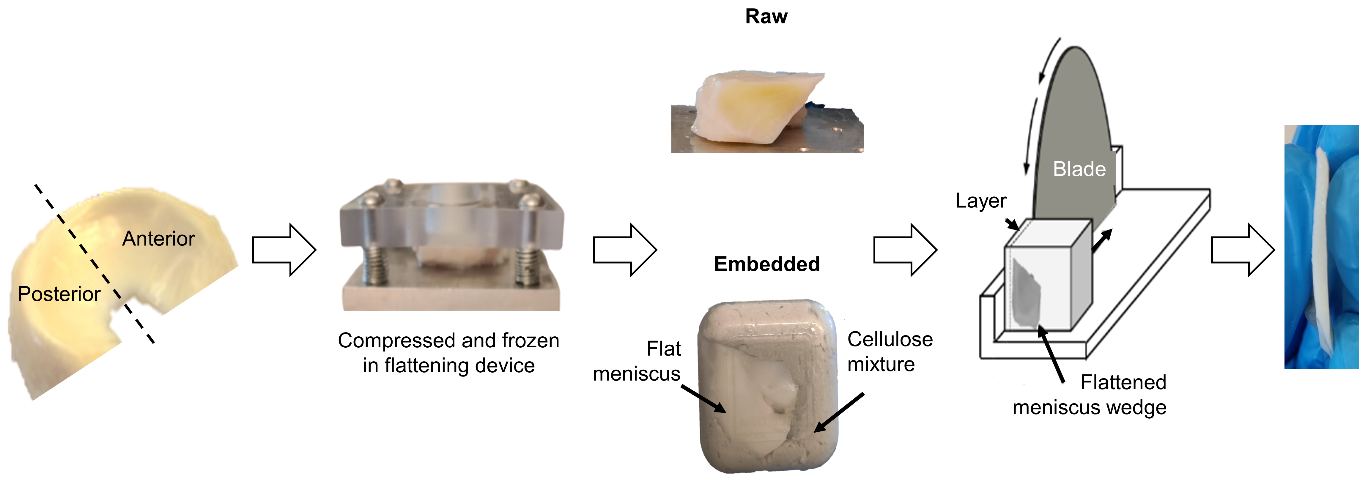


**Figure S1.** Layering process to achieve flatten meniscal plates for testing.


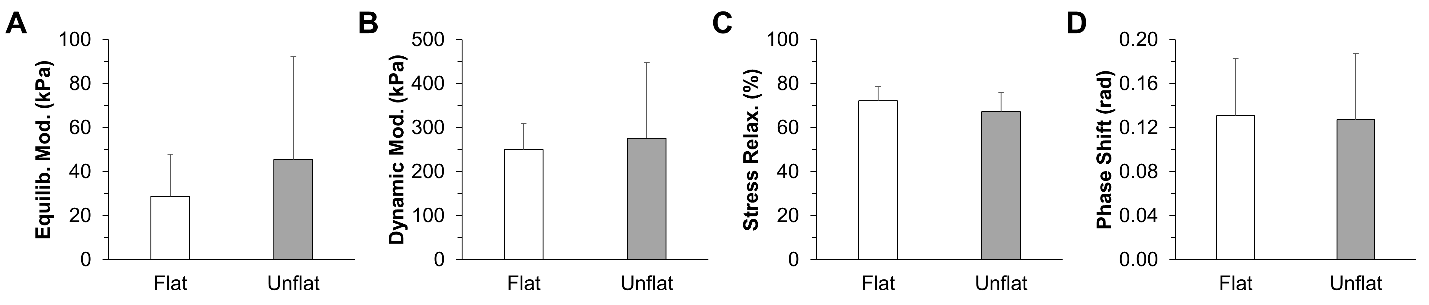


**Figure S2.** Compressive properties including A) equilibrium modulus, B) dynamic modulus, C) stress relaxation, and D) phase shift showed no statistically significant differences between flattened and unflattened meniscal tissue (*p* > 0.48).

## Validation of Pin-on-Plate Wear Testing Systems

A series of validation tests were conducted to ensure the accuracy of each pin-on-plate system. Linear translation accuracy was assessed by driving the rail carriage a known stroke length using the stepper motor. The horizontal displacement was measured using the stepper motor’s optical encoder feedback and verified with digital calipers (± 0.005 mm). Rotational accuracy was similarly assessed by having the servo motor rotate a set number of degrees. The angular displacement was recorded using the feedback from the motor’s position encoder and verified using a protractor (± 0.5°). The accuracy of the linear displacement sensor was determined by measuring a precision-calibrated block with known thickness. For each test, accuracy was defined as the absolute mean difference between measured and known values (|V_measured_ - V_actual_|), while error was defined as the absolute mean percentage error (100 * |V_measured_ - V_actual_|/V_actual_). Each validation test was conducted five times for every pin-on-plate system, with accuracy and error results shown in **Table S1**. It is important to note that there were no significant differences between pin-on-plate systems.

**Table S1.** Pin-on-plate system accuracy and error

|  | Linear Translation | | Rotation | | Vertical Displacement | |
| --- | --- | --- | --- | --- | --- | --- |
| Pin-on-Plate System No. | Accuracy (mm) | Error  (%) | Accuracy (deg) | Error  (%) | Accuracy (µm) | Error  (%) |
| 1 | 0.01 ± 0.01 | 0.27 ± 0.12 | 1.95 ± 1.17 | 1.97 ± 1.65 | 7.0 ± 4.0 | 1.15 ± 0.54 |
| 2 | 0.02 ± 0.01 | 0.40 ± 0.20 | 0.47 ± 0.70 | 0.52 ± 0.74 | 6.0 ± 4.0 | 0.94 ± 0.60 |
| 3 | 0.03 ± 0.01 | 0.53 ± 0.12 | 1.53 ± 0.66 | 1.62 ± 1.79 | 6.0 ± 3.0 | 1.11 ± 0.66 |

## Polarized Light Microscopy: Criteria for Measuring Superficial Layer Thickness

Polarized light microscopy (PLM) was utilized to identify and quantify the superficial layer thickness of meniscal tissue based on differences in birefringence. This optical property occurs due to the highly anisotropic collagen fiber structure, where regions with well-aligned fibers exhibit strong birefringence while areas with more randomly oriented fibers show reduced birefringence.^2^ Since the meniscal superficial layer consists of randomly oriented collagen fibers, it was therefore identified by its lack of birefringence, where a distinct band of reduced or absent birefringence was used to delineate the boundary between the superficial and inner layers **(Fig. S3)**.

As no standardized criteria exists for measuring this layer in meniscal tissue, to our knowledge, we developed a custom protocol to guide identification and measurement. Following successful imaging of all specimens (see Section 2.7), superficial layer thickness was quantified from PLM images using Leica Application Suite X software. A standardized measurement protocol was utilized for each specimen image (*n* = 51) where a total of five measurements were taken along the superficial layer: one central measurement and four additional measurements positioned ±1000 μm and ±2000 μm from the center. This spatial distribution was selected to account for potential regional variability in superficial layer thickness. To standardize the measurement environment, all thickness measurements were performed under consistent image contrast and zoom settings within the software.

Thickness measurements were taken perpendicular to the tissue surface, extending from the topmost surface to the interface identified by a pronounced increase in birefringence intensity **(Fig. S3; light band)**. Measurements were performed independently by three individuals with the images randomized and test conditions blinded. Intra-rater reliability was assessed by having all reviewers repeat the measurement process one week after the initial evaluation. Inter- and intra-rater agreement were evaluated using intraclass correlation coefficients (ICCs).^3^ Inter-rater agreement was moderate (ICC = 0.70) and intra-rater agreement ranged from 0.73 to 0.94. While this method allowed for consistent application across reviewers, moderate ICC values suggest some subjectivity remains in identifying the superficial boundary. Despite this, our measured thickness of the bovine meniscal superficial layer aligns reasonably well with the approximately 10 µm thickness reported for human meniscus,^4^ especially considering the larger bovine joint size.


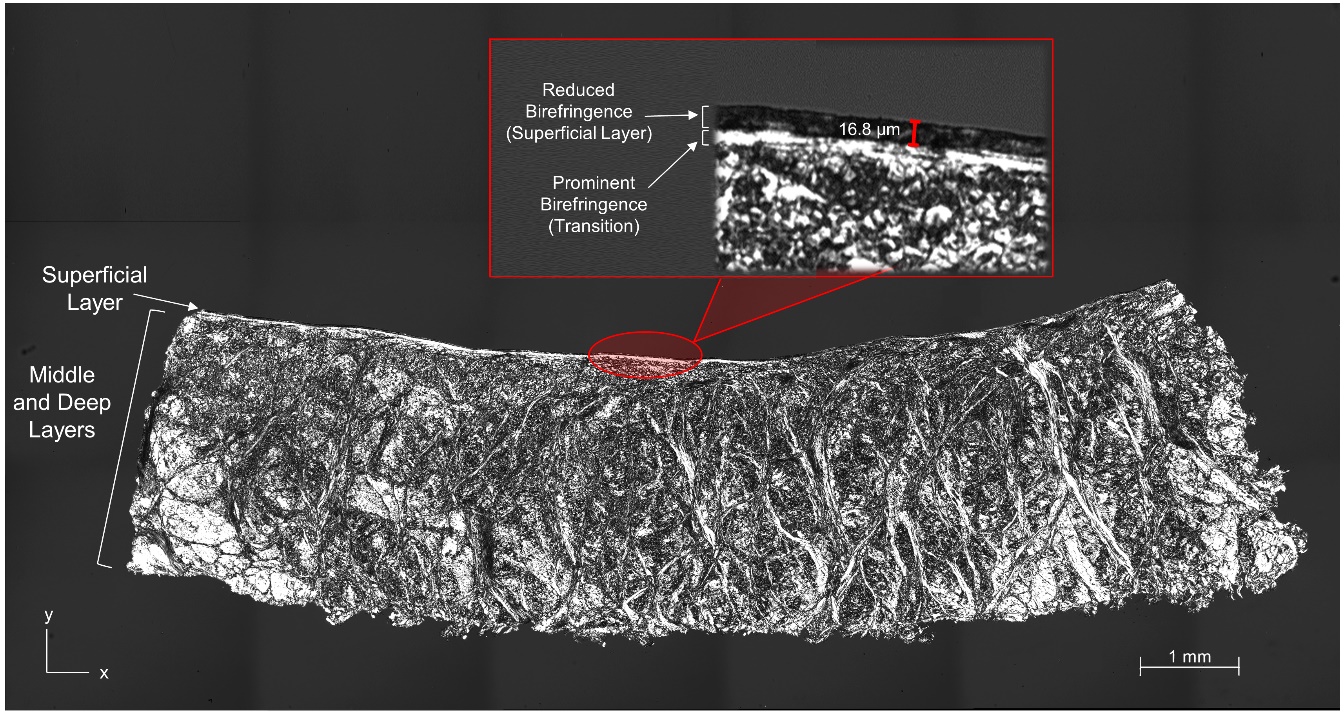


**Figure S3.** Polarized light image of meniscal tissue illustrating the superficial layer identified by its lack of birefringence (dark band). Thickness of the superficial layer (red bar) was measured perpendicular to the tissue surface, extending from the top edge to the prominent transition in birefringence (light band) that separates the superficial region from the underlying middle and deep layers.

## Raw Changes in Meniscal Compressive Properties

Compressive properties (equilibrium modulus, dynamic modulus, linear modulus, stress relaxation, relaxation rate, and phase shift) were evaluated before (Pre) wear testing and after (Post) the 24-hour recovery period. Average values are reported in **Table S2**.

**Table S2.** Compressive properties of meniscus specimens before and after wear testing for each of our main factors: motion type (load soak, unidirectional, multidirectional), loading magnitude (0, 0.5, 1.0, 1.5 MPa), and fiber orientation (longitudinal, transverse). Values reported as average ± standard deviation.

|  |  |  | *Equilib.*  *Mod. (kPa)* | | *Dyn.*  *Mod. (kPa)* | | *Linear*  *Mod. (kPa)* | | *Stress*  *Relax. (%)* | | *Relax.*  *Rate (s^-1^)* | | *Phase*  *Shift (rad)* | |
| --- | --- | --- | --- | --- | --- | --- | --- | --- | --- | --- | --- | --- | --- | --- |
|  |  |  |  | |  | |  | |  | |  | |  | |
| Motion  Type | Load Soak | Pre | 477 ± 318 | | 2699 ± 1749 | | 1899 ± 1538 | | 55.0 ± 9.1 | | 0.010 ± 0.005 | | 0.16 ± 0.05 | |
|  |  | Post | 524 ± 327 | | 3237 ± 1856 | | 2750 ± 1757 | | 61.9 ± 8.3 | | 0.010 ± 0.006 | | 0.16 ± 0.04 | |
|  |  |  |  | |  | |  | |  | |  | |  | |
|  | Uni | Pre | 599 ± 418 | | 2867 ± 1870 | | 1998 ± 1421 | | 49.4 ± 8.6 | | 0.008 ± 0.010 | | 0.14 ± 0.04 | |
|  |  | Post | 581 ± 381 | | 2981 ± 1722 | | 2483 ± 1631 | | 54.8 ± 8.7 | | 0.011 ± 0.008 | | 0.14 ± 0.03 | |
|  |  |  |  | |  | |  | |  | |  | |  | |
|  | Multi | Pre | 520 ± 280 | | 2474 ± 1817 | | 1822 ± 1547 | | 48.1 ± 11.3 | | 0.007 ± 0.008 | | 0.13 ± 0.04 | |
|  |  | Post | 522 ± 298 | | 2850 ± 1740 | | 2547 ±1946 | | 56.2 ± 11.3 | | 0.012 ± 0.011 | | 0.15 ± 0.05 | |
|  |  |  |  |  |  |  |  |  |  |  |  |  |  |  |
| Loading Magnitude | 0 MPa | Pre | 510 ± 244 | | 2695 ± 1847 | | 1708 ± 1139 | | 51.3 ± 7.7 | | 0.007 ± 0.003 | | 0.13 ± 0.03 | |
|  |  | Post | 537 ± 294 | | 2909 ± 2188 | | 2097 ± 1660 | | 52.2 ± 6.4 | | 0.006 ± 0.002 | | 0.14 ± 0.01 | |
|  |  |  |  | |  | |  | |  | |  | |  | |
|  | 0.5 MPa | Pre | 578 ± 419 | | 2684 ± 2090 | | 1871 ± 1691 | | 47.1 ± 10.0 | | 0.008 ± 0.012 | | 0.14 ± 0.04 | |
|  |  | Post | 567 ± 409 | | 2849 ± 1950 | | 2225 ± 1750 | | 51.4 ± 10.0 | | 0.010 ± 0.010 | | 0.15 ± 0.05 | |
|  |  |  |  | |  | |  | |  | |  | |  | |
|  | 1.0 MPa | Pre | 592 ± 296 | | 3210 ± 1849 | | 2321 ± 1494 | | 51.8 ± 10.8 | | 0.009 ± 0.010 | | 0.14 ± 0.04 | |
|  |  | Post | 620 ± 303 | | 3481 ± 1701 | | 3240 ± 2020 | | 58.3 ± 10.0 | | 0.014 ± 0.012 | | 0.14 ± 0.02 | |
|  |  |  |  | |  | |  | |  | |  | |  | |
|  | 1.5 MPa | Pre | 509 ± 351 | | 2119 ± 1436 | | 1539 ± 1150 | | 47.4 ± 8.9 | | 0.007 ± 0.005 | | 0.13 ± 0.03 | |
|  |  | Post | 467 ± 296 | | 2416 ± 1356 | | 2080 ± 1354 | | 56.7 ± 9.3 | | 0.010 ± 0.007 | | 0.15 ± 0.04 | |
|  |  |  |  |  |  |  |  |  |  |  |  |  |  |  |
| Fiber Orientation | Long. | Pre | 510 ± 293 | | 2884 ± 2093 | | 2128 ± 1703 | | 53.4 ± 9.3 | | 0.009 ± 0.008 | | 0.14 ± 0.03 | |
|  |  | Post | 547 ± 326 | | 3122 ± 1903 | | 2868 ± 2010 | | 59.8 ± 9.2 | | 0.012 ± 0.009 | | 0.15 ± 0.04 | |
|  |  |  |  | |  | |  | |  | |  | |  | |
|  | Trans. | Pre | 609 ± 407 | | 2457 ± 1551 | | 1693 ± 1195 | | 44.1 ± 8.4 | | 0.006 ± 0.010 | | 0.13 ± 0.04 | |
|  |  | Post | 557 ± 360 | | 2709 ± 1515 | | 2162 ± 1466 | | 51.1 ± 9.1 | | 0.011 ± 0.010 | | 0.15 ± 0.04 | |
|  |  |  |  | |  | |  | |  | |  | |  | |

## Stress-Strain Curves

The full stress–strain curves for each testing condition are presented in **Figure S4**. The linear modulus was calculated as the linear fit between 10% and 12% strain using a custom MATLAB script. A representative fit can be seen in **Figure S5** for a multidirectional, 1.0 MPa, transverse case.


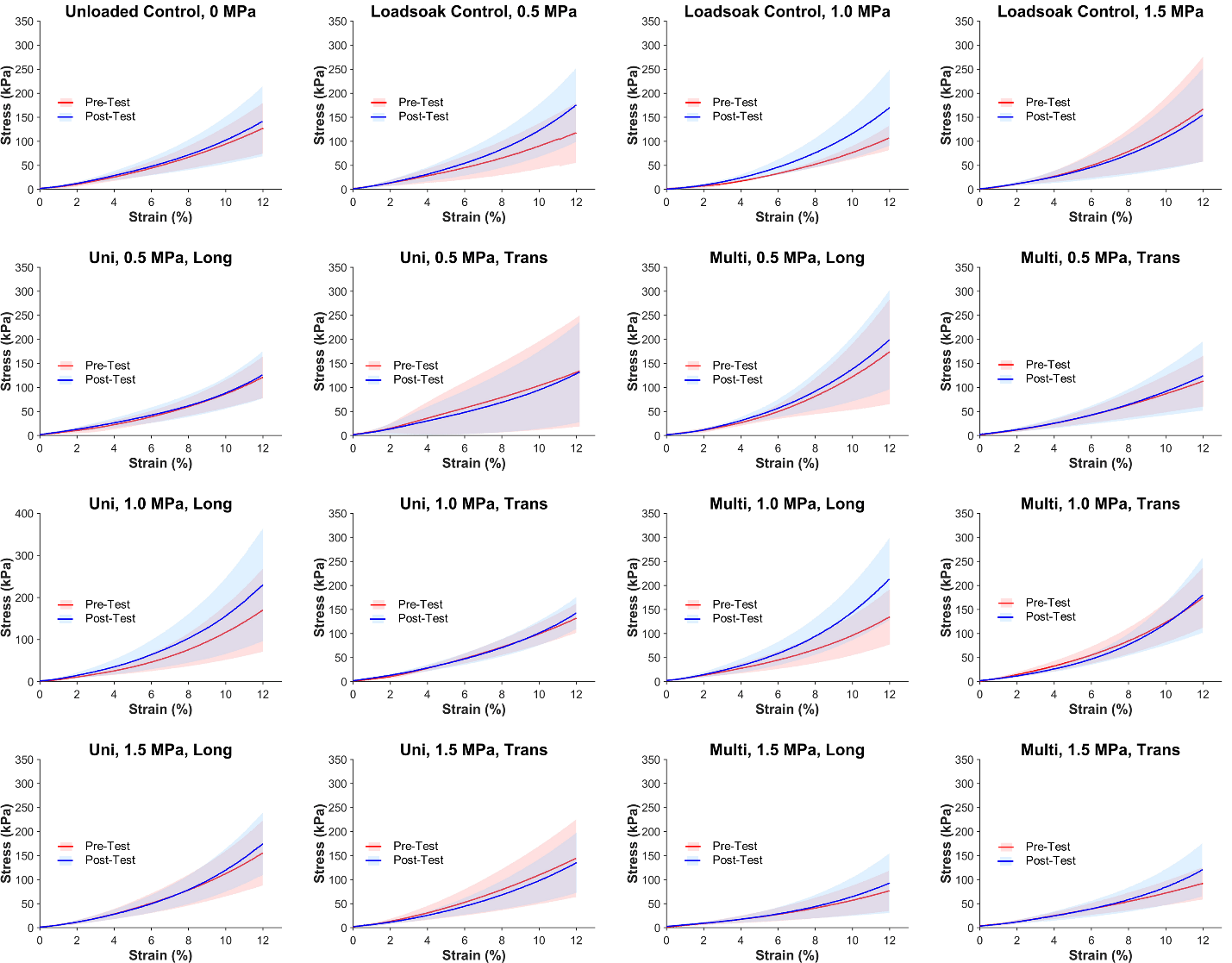


**Figure S4.** Stress–strain curves for each testing condition showing the average nonlinear behavior with ±95% shaded confidence intervals for pre- and post-testing.


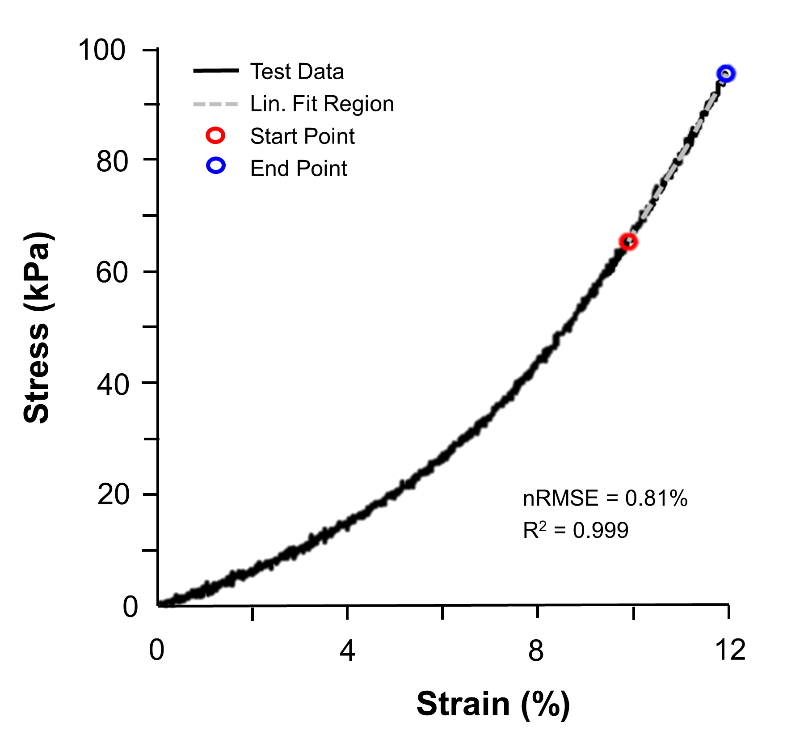


**Figure S5.** Representative test case illustrating the linear modulus fit between 10% and 12% strain resulting in a modulus value of 1450 kPa.

## Meniscal Fraying

Representative brightfield images of the superficial meniscal surface from each wear and control group are shown in Fig. S6. Each image was selected to reflect the average fraying grade for its respective testing group, where the inset value indicates the fraying grade measure for the specific specimen. These examples illustrate the extent of fraying across groups and qualitatively support the quantitative grading results **(Fig. 6G)**.


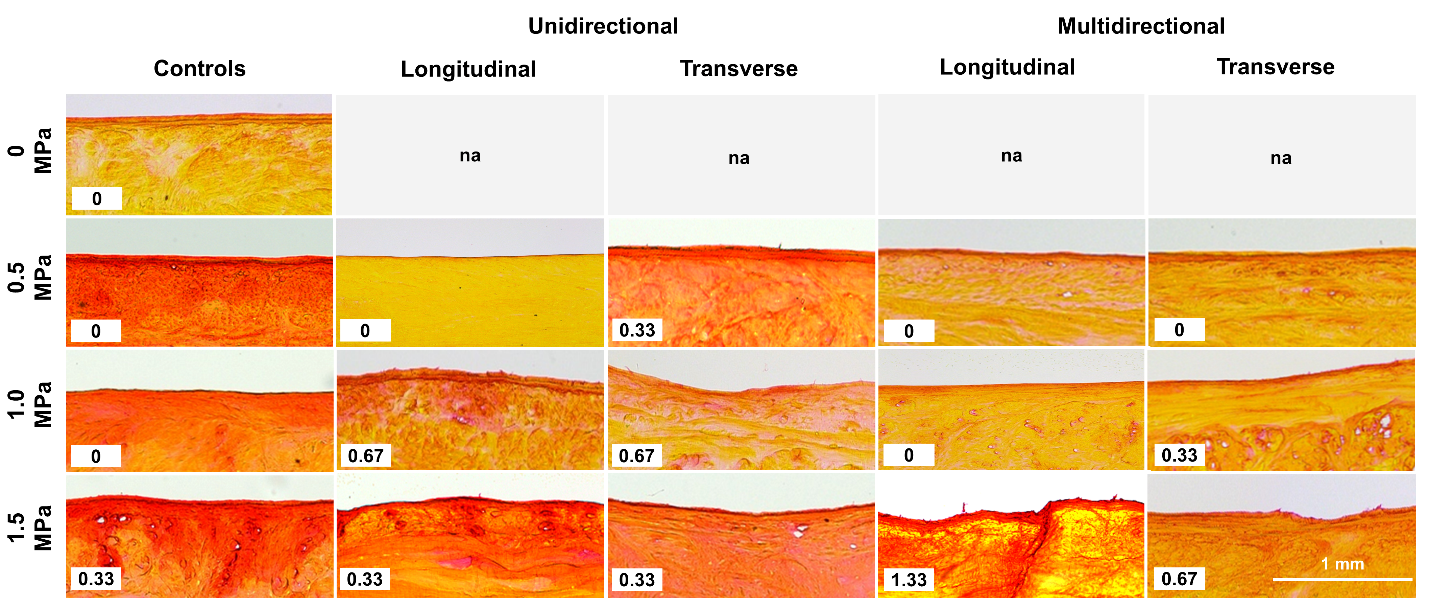


**Figure S6.** Representative color brightfield images of all wear and control testing groups. The inset number denotes the average specimen fraying grade measured for that shown image, which is a close approximation of the mean fraying grade calculated for that image’s respective test group (Fig. 6G).

## Cartilage Pin Height Loss

The cartilage pin remained largely intact with no prominent surface fraying following testing **(Fig. S7)**. The unidirectional and multidirectional wear tests did have significantly more loss in cartilage pin height compared to the load soak control tests (*p* < 0.01), however, no significant differences in the loss of cartilage pin height were found between wear testing groups: motion type (*p* = 0.16), loading magnitude (*p* = 0.20), fiber orientation (*p* = 0.38) **(Fig. S8)**. Additionally, the mean loss in cartilage pin height was 0.063 ± 0.09 mm (1.3%), which is approximately a third of the mean loss measured in meniscal thickness. Further, the loss of cartilage pin height and the loss of meniscal volume were poorly correlated (*r* = 0.23, *p* = 0.06), indicating that wear of the cartilage pin did not substantially contribute to meniscal volume loss in this study.


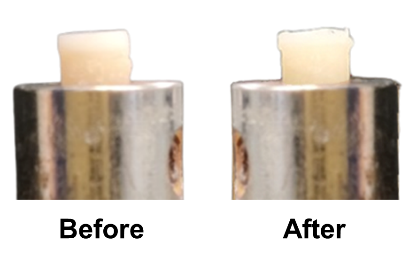


**Figure S7.** Representative cartilage pin from a unidirectional, 1.0 MPa, longitudinal test showing minimal height loss and little to no surface damage.


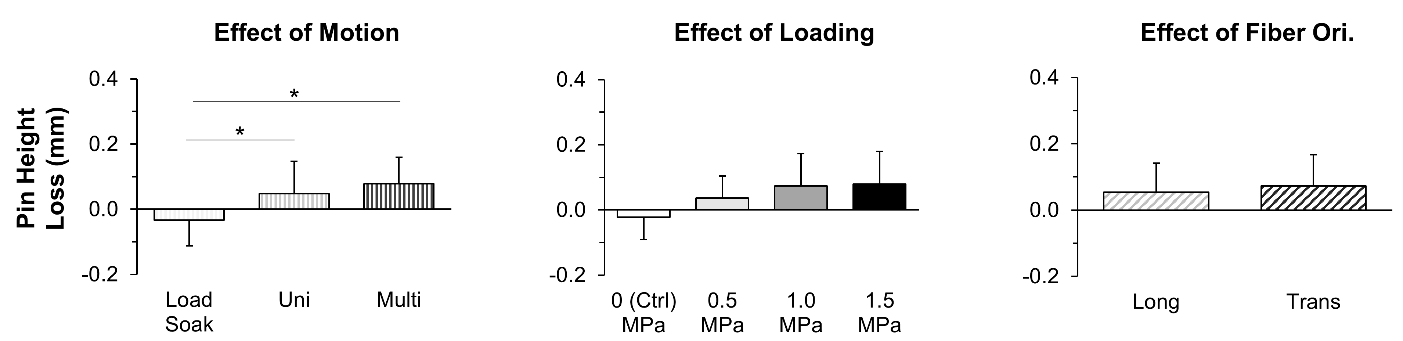


**Figure S8.** Effects of motion type, loading magnitude, and fiber orientation on cartilage pin height loss (post-pre).

## Supplemental Material References

1. Wale ME, Nesbitt DQ, Henderson BS, Fitzpatrick CK, Creechley JJ, Lujan TJ. Applying ASTM Standards to Tensile Tests of Musculoskeletal Soft Tissue: Methods to Reduce Grip Failures and Promote Reproducibility. *J Biomech Eng*. 2021;143(1):011011. doi:10.1115/1.4048646

2. Changoor A, Tran-Khanh N, Méthot S, et al. A polarized light microscopy method for accurate and reliable grading of collagen organization in cartilage repair. *Osteoarthritis Cartilage*. 2011;19(1):126-135. doi:10.1016/j.joca.2010.10.010

3. Pauli C, Grogan SP, Patil S, et al. Macroscopic and histopathologic analysis of human knee menisci in aging and osteoarthritis. *Osteoarthritis and Cartilage*. 2011;19(9):1132-1141. doi:10.1016/j.joca.2011.05.008

4. Petersen W, Tillmann B. Collagenous fibril texture of the human knee joint menisci. *Anat Embryol*. 1998;197(4):317-324. doi:10.1007/s004290050141
